# Cancer cell employs a microenvironmental neural signal *trans*-activating nucleus-mitochondria coordination to acquire stemness

**DOI:** 10.1038/s41392-023-01487-4

**Published:** 2023-07-19

**Authors:** Bin He, Rui Gao, Shasha Lv, Ailin Chen, Junxiu Huang, Luoxuan Wang, Yunxiu Feng, Jiesi Feng, Bing Liu, Jie Lei, Bing Deng, Bin He, Bai Cui, Fei Peng, Min Yan, Zifeng Wang, Eric W-F Lam, Bilian Jin, Zhiming Shao, Yulong Li, Jianwei Jiao, Xi Wang, Quentin Liu

**Affiliations:** 1 State Key Laboratory of Oncology in South China, Sun Yat-sen University Cancer Center, Guangzhou, 510060 PR China; 2 Department of Medical Oncology, The Seventh Affiliated Hospital, Sun Yat-sen University, Shenzhen, 510275 PR China; 3 Institute of Cancer Stem Cell, Cancer Center, Dalian Medical University, Dalian, 116023 PR China; 4 Zhongshan School of Medicine, Sun Yat-sen University, Guangzhou, 510080 PR China; 5 State Key Laboratory of Membrane Biology, Peking University School of Life Sciences, Beijing, 100871 PR China; 6 Department of Surgery and Cancer, Imperial College London, London, W12 0NN UK; 7 Department of Breast Surgery, Precision Cancer Medicine Center, Fudan University Shanghai Cancer Center, Shanghai, 200032 PR China; 8 State Key Laboratory of Stem Cell and Reproductive Biology, Institute of Zoology, Chinese Academy of Sciences, Beijing, 100101 PR China

**Keywords:** Cancer microenvironment, Cancer stem cells, Genome informatics, Stem-cell niche

## Abstract

Cancer cell receives extracellular signal inputs to obtain a stem-like status, yet how tumor microenvironmental (TME) neural signals steer cancer stemness to establish the hierarchical tumor architectures remains elusive. Here, a pan-cancer transcriptomic screening for 10852 samples of 33 TCGA cancer types reveals that cAMP-responsive element (CRE) transcription factors are convergent activators for cancer stemness. Deconvolution of transcriptomic profiles, specification of neural markers and illustration of norepinephrine dynamics uncover a bond between TME neural signals and cancer-cell CRE activity. Specifically, neural signal norepinephrine potentiates the stemness of proximal cancer cells by activating cAMP-CRE axis, where ATF1 serves as a conserved hub. Upon activation by norepinephrine, ATF1 potentiates cancer stemness by coordinated *trans*-activation of both nuclear pluripotency factors MYC/NANOG and mitochondrial biogenesis regulators NRF1/TFAM, thereby orchestrating nuclear reprograming and mitochondrial rejuvenating. Accordingly, single-cell transcriptomes confirm the coordinated activation of nuclear pluripotency with mitochondrial biogenesis in cancer stem-like cells. These findings elucidate that cancer cell acquires stemness via a norepinephrine-ATF1 driven nucleus-mitochondria collaborated program, suggesting a spatialized stemness acquisition by hijacking microenvironmental neural signals.

## Introduction

Cancer stemness, characterized by the property to clonogenicity, self-renewal and multilineage differentiation, is currently a functionally defined, plastic cellular status.^1,2^ The plastic nature of cancer stem-like status suggests that extracellular inputs are required to instruct stemness via a complex interplay between cell intrinsic and extrinsic factors. Pluripotent transcription factors, which control the fate of normal stem cells, are core intrinsic regulators of cancer stem-like cells (CSCs).^3^ Transcription factors such as MYC drives stemness in a variety of cancers including breast^4^ and pancreatic^5^ cancer. Extracellular cytokines from tumor microenvironment (TME), such as TGF-β, IL-6 are sufficient to initiate stem-like status.^2,6^ Among the TME factors, neural signals have increasingly been recognized to facilitate cancer progression.^7–9^ For instance, norepinephrine,^10^ gamma-aminobutyric acid^11^ and glutamate^12,13^ have been found to enable invasive cancer phenotype, which is indicative of cancer stem-like properties. Indeed, TME neural signals are recently appreciated as a signal hub linking psychological state and cancer progression.^8^ However, little is known about how TME localized neural signals are integrated with cell-intrinsic stemness factors to establish cancer stem-like state.

Neurotransmitters are well-appreciated neural signals involved in cancer development.^8,9^ Among the neurotransmitters, adrenergic signaling induced norepinephrine plays a central role in cancer progression.^9,10,14,15^ Norepinephrine activated adrenergic receptors modulate the activity of transmembrane adenylyl cyclase, which generates 3’,5’-cyclic adenosine monophosphate (cAMP) to control cellular response to neural signals.^9,16^ The transcriptional response to cAMP is primarily dependent on protein kinase A (PKA), which phosphorylates and activates cAMP responsive element (CRE) binding transcription factors CREB/ATF1 (activating transcription factor 1) to control target gene transcription.^17–20^ The CRE binding transcription factors are thus crucial for cellular response to chronic stress-induced adrenergic neurotransmitters. Yet how adrenergic signaling induced CRE signaling contributes to cancer stemness remains poorly understood. The finding that CDK5-CREB1 enhances glioma stem cells and AMPK-CREB1 potentiates glioblastoma stem cells indicate the role of cAMP responsive program in stemness.^21,22^ A recent study in metastatic colorectal cancer confirms that cAMP-CREB1 signaling enhances cancer stemness.^23^ However, whether cancer cell acquires stemness through employing a TME neural signal dependent CRE program remains undetermined.

Here, we characterize pan-cancer TME responsive stemness transcriptional factors based on the stemness transcription profiles from 10852 tumors of 33 TCGA cancer types and 17 pairs of adherent-oncosphere in 11 cancer types. Among the TME responsive stemness factors, CRE factors are conservatively enriched and required for stem-like function in multiple cancer types. Deconvolution of cell compositions, immunofluorescence and time-lapse fluorescent imaging in tumors reveal a connection between TME neural signal and cancer-cell CRE activity. Specifically, microenvironmental norepinephrine, an adrenergic neurotransmitter that is activated by chronic stress, potentiates the stemness activity of proximal cancer cells through cAMP-ATF1 axis. ATF1 integrates adrenergic signal and cell-intrinsic stemness factors by simultaneously coordinated *trans*-activation of genes involved in both nuclear pluripotency and mitochondrial rejuvenation, thereby driving cancer cells into stem-like state.

## Results

### CRE is a convergent TME-responsive activator of CSCs

**Fig. 1:**
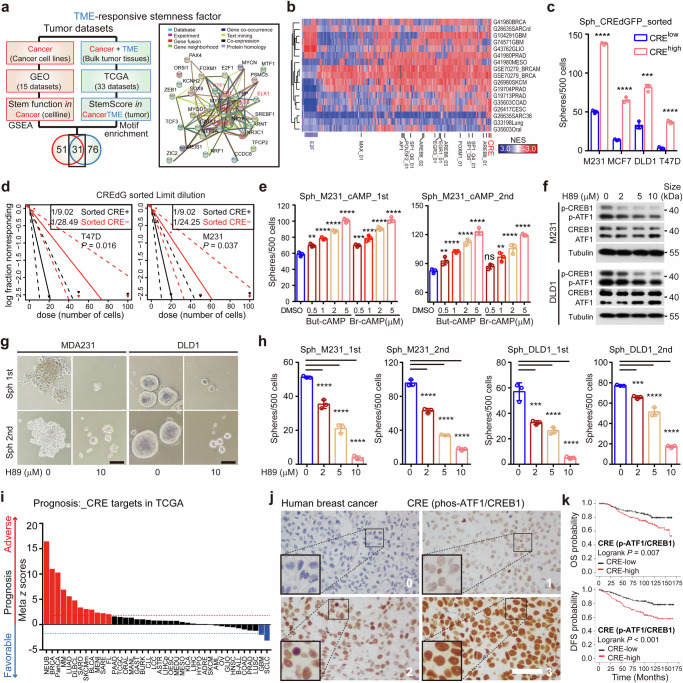
CRE is a pan-cancer TME responsive stemness effector. **a** A scheme to screen TME responsive transcriptional regulators of CSCs. Gene set enrichment analysis (GSEA) in paired sphere-adherent cells identifies CSC-enriched transcription factors. Transcription factor motif analysis of genes enriched in StemnessScore^high^ tumors identified TME responsive stemness factors in vivo. The STRING (http://string-db.org/) interaction network of 31 TME responsive stemness transcription factors overlapped in GSEA/TCGA datasets was present in the right panel. CRE associated factors were labeled in red in the network. **b** Normalized enrichment scores (NES) for top transcription factors enriched in 17 paired sphere-adherent from 15 independent datasets. NES was determined by GSEA. CRE, cAMP responsive element. **c** Sphere formation of sorted cells (MDA-MB-231, MCF-7, DLD1 and T47D) according to the CRE-responsive GFP reporter (CRE-dGFP) activity (*n* = 3; mean ± SD; *P* values, Tukey’s multiple comparisons after 1-way ANOVA). **d** In vitro limit dilution assay of sorted CRE-dGFP^+^ and CRE-dGFP^-^ populations in MDA-MB-231 and T47D cells. Differences in stem cell frequencies were determined by ELDA (https://bioinf.wehi.edu.au/software/elda/). *n* = 6 for each group. **e** Primary (left panel) and secondary (right panel) sphere formation of MDA-MB-231 cells in indicated doses of cAMP mimics in sphere media (Butyl-cAMP and 8-Br-cAMP, *n* = 3; mean ± SD; *P* values, Tukey’s multiple comparisons after 1-way ANOVA). **f** Western blot analysis of MDA-MB-231 (upper panel) and DLD1 cells treated with PKA inhibitor H89 for 1 hr. Cell extracts were analyzed with antibodies against phospho-CREB1/ATF1, total ATF1/CREB1 and alpha-tubulin. **g** Representative images of sphere formation in MDA-MB-231 (left panel) and DLD1 (right panel) cells treated with H89 in sphere media. Scale bar represents 60 μm. **h** Primary and secondary sphere formation of MDA-MB-231 (left panel) and DLD1 (right panel) cells treated with H89 in sphere media (*n* = 3; mean ± SD; *P* values, Tukey’s multiple comparisons after 1-way ANOVA). **i** The prognostic meta-*z* sores of ATF1/CREB1 target genes (collected form MSigDB, Supplementary Table S2) among cancer types. Meta-*z* scores were calculated by unweighted prognostic *z* scores of individual genes of the signature. **j** Representative IHC staining of phospho-CREB1/ATF1 in breast cancer specimens. Numbers indicate phospho-CREB1/ATF1 expression scores. Scale bar represents 50 μm. **k** Kaplan Meier curves of estimated overall survival (OS, left panel) and disease-free survival (DFS, right panel) of breast cancer patients with low (*n* = 238) and high (*n* = 128) phospho-CREB1/ATF1 levels (*P* values, log-rank test). *P < 0.05; **P < 0.01; ***P < 0.001; ****P < 0.0001; ns not significant

The central role of transcription factors in TME signal-responsive processes prompts us to characterize TME responsive stemness programs from transcription factors that are specialized to utilize TME signals. The previous report that machine learning-based StemnessScores corelate with tumor immune microenvironment indicates that StemnessScores are indicative of TME responsive stemness programs.^24^ We developed a strategy to identify the TME responsive stemness programs by integrating both in vivo TME responsive stemness programs and function-based cancer cell intrinsic stemness factors (Fig. 1a). Single sample gene set enrichment analysis (ssGSEA)^25,26^ in The Cancer Genome Atlas (TCGA) dataset revealed the landscape of correlations between StemnessScores and tumor microenvironmental cells. StemnessScores are positively correlated with neural cells and negatively correlated with immune cells across multiple cancer types, respectively (Supplementary Fig. S1a). We thus characterized the transcriptional profiles of 10852 intact tumors of 33 TCGA cancer types to define transcription factors that control genes highly expressed in StemnessScore^high^ tumors as 107 TME associated stemness factors (Supplementary Table. S1a, *P*-value < 0.01, FDR *q* < 0.05).

To identify the TME stemness factors that are functionally involved in cancer stemness, we reanalyzed CSC regulators from oncosphere studies.^4,27,28^ Indeed, sphere-forming capacity predicts in vivo CSC frequencies in 30 cell lines, confirming the enrichment of functional stemness in oncospheres (Supplementary Fig. S1b, Pearson *r* = 0.6087, *P* = 0.0004). The group of functional cancer stemness factors were characterized based on the gene set enrichment analyses (GSEA)^29^ in 17 pairs of transcriptional profiles of oncosphere enriched CSCs among 11 cancer types from the Gene Expression Omnibus (GEO, Supplementary Table. S1b, GSEA normal *P*-value < 0.05). Enrichment of glioblastoma stem cell-specific gene sets in glioblastoma spheres verifies the capacity of the GSEA approach to identify functional stemness factors (Supplementary Fig. S1c). The 31 of the 82 functional stemness factors that overlaps with the 107 TME associated stemness factors were defined as TME responsive stemness factors (Fig. 1a). Among the 31 TME responsive stemness factors, cAMP responsive factors (ATF1, CREB1, JUN, FOS, SP1 and ELK1) are convergently enriched in oncosphere defined stem-like cells (Fig. 1a, b and Supplementary Fig. S1d, e). Indeed, transcription factors binding cAMP responsive element (CRE) motif (ATF1 and CREB1) and CRE-like motif (AP1) are enriched in the oncospheres of multiple cancer types (7/11 and 6/11 of cancer types for ATF1/CREB1 and AP1, respectively, Fig. 1b). These findings suggest that CRE is a conserved TME responsive cancer stemness program.

To examine whether CRE activity is required for cancer stemness, we constructed a transcriptional reporter with a destabilized GFP (dGFP) driven by a synthetic CRE promoter (CRE-dGFP), which faithfully report CRE activity under cAMP stimulators (Butyl-cAMP and Forskolin) or inhibitors (SQ22536, Supplementary Fig. S2a). Increased proportion of CRE-dGFP^+^ cells is detected in spheres compared to adherent cells, indicating higher CRE activity in CSCs (Supplementary Fig. S2b). To determine the stemness in CRE-dGFP cells, breast (MDA-MB-231, T47D and MCF-7), lung (H460) or colon (DLD1) cancer cells were sorted into CRE^high^ or CRE^low^ subsets according to their CRE-dGFP intensity. The frequencies of sphere-forming cells are significantly higher in the CRE^high^ subsets compared with CRE^low^ cells in candidate cell lines (Fig. 1c, d and Supplementary Fig. S2c). To assess the effect of CRE activity on stemness, cell-permeable cAMP mimics (8-Br-cAMP or Dibutyryl-cAMP)^30,31^ are applied to activate CRE activity (Supplementary Fig. S2d). The cAMP mimics enhance the sphere-forming efficiency in MDA-MB-231, BT-549, DLD1 and H460 cells, whereas cell proliferation is mildly affected by the same does of cAMP mimics in both MDA-MB-231 and BT-549 cells (Fig. 1e and Supplementary Fig. S2e–g). A protein kinase A (PKA) inhibitor H89,^32^ which blocks the cAMP-induced activation of CREB1 and ATF1 transcription factors, is applied to suppress CRE activity (Fig. 1f and Supplementary Fig. S2h). Consistently, sphere formation is impaired by H89 in MDA-MB-231, T47D, MCF-7, BT-549, A549 and DLD1 cells (Fig. 1g, h and Supplementary Fig. S2i, j). In contrast, cell proliferation is modestly affected by these doses of H89 in MDA-MB-231, T47D and DLD1 cells (Supplementary Fig. S2k). Another PKA inhibitor Metadoxine^33^ and the MSK1/2 inhibitor SB-747651A,^34^ which also inhibit the phosphorylation of CREB1/ATF1,^35^ suppress the sphere formation of breast (BT-549, MDA-MB-231) and lung (A549) cancer cells (Supplementary Fig. S2l–n). These data demonstrated that CRE activity is required for cancer stemness.

Next, we examined the clinical relevance of CRE activity by examining the prognostic scores of CRE target genes in the PRECOG dataset.^36^ Core CRE target genes (43 ATF1/CREB1 targets listed in Supplementary Table S2) are associated with adverse prognoses in 12/39 of cancer types, whereas favorable prognoses are observed in 2/39 of the cancer types (Fig. 1i). To confirm the clinical significance of CRE activity, the levels of activated CRE factors (phospho-CREB1^S133^/ATF1^S63^)^37^ are determined in a cohort of breast cancer patients (Fig. 1j). In agreement, high phospho-CREB1/ATF1 intensity is associated with poor overall survival and disease-free survival in breast cancer (*P* = 0.007 and *P* < 0.001, respectively, Fig. 1k). Moreover, Cox repression indicates that phospho-CREB1/ATF1 intensity is an independent factor in determining disease-free status in breast cancer (*P* < 0.001, Table 1). Among the clinical parameters, CRE activity is positively correlated with negative HER2 status and distant metastasis (*P* = 0.020 and 0.002, respectively, Supplementary Table S3). These findings indicate that CRE activity indicates stemness, recurrence and poor clinical prognosis in cancer.

**Table 1:** Cox regression of phospho-CREB1/ATF1 in breast cancer (DFS, *n* = 367)

	DFS in entire cohort			
	Univariate		Multivariate	
	*P*	HR	95%CI	*P*
ER (positive)	0.939			
PR (positive)	0.655			
HER2 (positive)	0.04*	1.935	1.279–2.925	0.002*
Age (>50)	0.625			
Perimenopauses	0.338			
Tumor size (>2 cm)	0.002*			
Node status (positive)	<0.001*	1.582	0.938–2.667	0.086
Tumor grade (III)	0.173			
Stage (III)	<0.001*	2.664	1.663–4.268	<0.001*
ATF1 (positive)	<0.001*	2.474	1.594–3.839	<0.001*

*Statistically significant values (*P* < 0.05)

### TME adrenergic signal directs CRE dependent cancer stemness

**Fig. 2:**
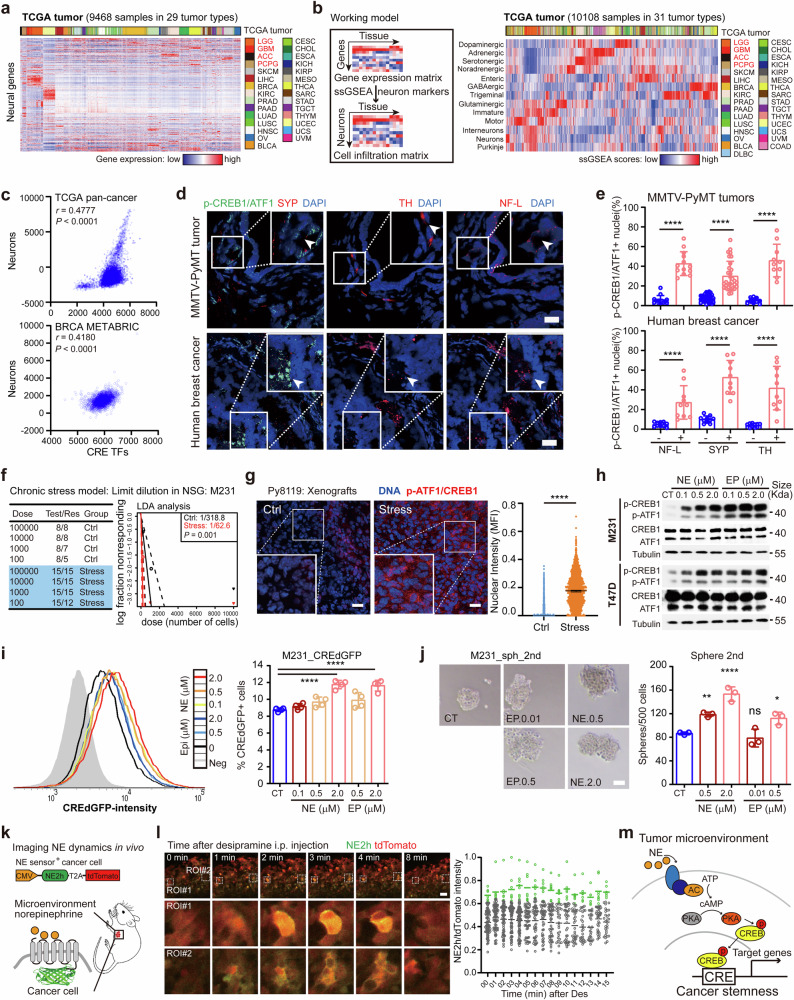
Proximal neural signals activate CRE-dependent stemness. **a** Heatmap showing the expressions of neural specific genes in TCGA tumors. Sidebar, tumors types. **b** Left panel, single sample GSEA (ssGSEA) based deconvolution of tissue associated nerves based on the gene expression profiles from the bulk RNA-seq data by neural specific marker gene set based analysis in individual tumors. Right panel, The neural signals in TCGA tumors according to the ssGSEA enrichment scores. **c** Correlation of CRE activity with neural signals in the pan-cancer (TCGA, upper panel) and METABRIC (breast cancer, lower panel) tumors. Correlations (Pearson *r*) between CRE and specific neural signals were determined according to their ssGSEA scores in individual tumors (*P* values, Pearson correlation). **d** Immunofluorescent analysis of phospho-CREB1/ATF1 intensity (green), neural markers (NF-L, TH and SYP, red) in mouse MMTV-PyMT (upper panel) and human (lower panel) breast tumors. Scale bar, 20 μm. **e** Nuclear phospho-CREB1/ATF1 intensity indicated by the percentages of phospho-ATF1/CREB1 positive nuclei per field (0.01 mm^2^ within neural marker positive area, *n* = 10) in mouse (upper panel) and human (lower panel) breast tumors, respectively. Neural signal negative area (*n* = 10) was used as controls (mean ± SD; 2-sided *t* test). **f** In vivo limit dilution assay of MDA-MB-231 cells in control or chronic stressed NSG mice. Differences in stem cell frequencies were determined by ELDA (https://bioinf.wehi.edu.au/software/elda/). *n* = 8 and 15 for control and stress groups, respectively. **g** Representative immunofluorescent staining of phospho-CREB1/ATF1 in Py8119 xenografts from control (Ctrl) and stressed (stress) mice (left panel). Quantification of nuclear phospho-CREB1/ATF1 intensity (median fluorescent intensity, MFI) in immunofluorescent Py8119 xenografts (right panel, *n* = 1295 and 1476 for control and stressed groups; mean ± SD; 2-sided *t* test). Scale bar, 20 μm. **h** Western blot analysis of MDA-MB-231 (upper panel) and T47D (lower panel) cells treated with epinephrine (EP) and norepinephrine (NE) for 30 min. Cell extracts were analyzed with phospho-CREB1/ATF1, total ATF1/CREB1 and Alpha-tubulin antibodies. **i** Left panel, flow cytometry analysis of CRE-dGFP reporter activity of MDA-MB-231 cells in response to 24 hr NE/EP treatment. Parental MDA-MB-231 cells without reporter transfection (Neg) were used as control. Right panel, percentage of CRE-dGFP^+^ cells in MDA-MB-231 in response to NE/EP treatment (24 hr). **j** Secondary sphere formation of MDA-MB-231 cells in the presence of NE/EP. Representative images of spheres were present. Scale bar, 60 μm. (for 2i–j, *n* = 4; mean ± SD; *P* values, Tukey’s multiple comparisons after 1-way ANOVA). **k** Schematic illustration of the experimental design for the time-lapse imaging of norepinephrine activity in the tumor microenvironment using fluorescent norepinephrine reporter (GRAB_NE2h_). **l** Left panel, representative images of GRAB_NE2h_ (green) and tdTomato (red) activity in the MDA-MB-231 xenograft before (0 min) and after (1-4 min) i.p. injection of the norepinephrine transporter (NET) blocker desipramine (10 mg/kg). Scale bar, 60 μm. Right panel, median fluorescent intensity of GRAB_NE2h_ fluorescence (upper) and tdTomato (lower) in the MDA-MB-231 xenograft following treatment with the desipramine (10 mg/kg). *n* = 75 cells from 3 mice for each condition. **m** Model of TME NE dependent cAMP responsive program that acts as a conserved mechanism driving cancer stemness. *P < 0.05; **P < 0.01; ***P < 0.001; ****P < 0.0001; ns not significant

Among the extracellular molecules, neural signals, which are positively correlated with StemnessScores (Supplementary Fig. S1a), serve as critical modulators of CRE activity.^16,17^ Based on the evidence that neural fibers contain abundant mRNAs,^38,39^ we speculate that neuron specific transcripts in bulk RNA-seq profiles of intact tissues may indicate localized neural compositions. Indeed, neuron specific transcripts are detected in the RNA-seq profiles of both normal tissues (12 tissues in the Genotype-Tissue Expression dataset, GTEx) and solid tumors (29 solid tumors in the TCGA). Enrichment of neural transcripts in brain tissues from both normal and tumor cohorts is consistent with the abundant neural signals in the brain (Fig. 2a and Supplementary Fig. S3a). To test whether cancer cell derived transcripts might contribute to the neuron specific transcripts in the bulk tumor transcriptome, we examined single-cell transcriptomic datasets of cancer cells. Indeed, rare neuron specific transcripts are detected in single cancer cells from melanoma, glioma, breast, cervical and pancreatic cancer, indicating that cancer-intrinsic transcripts are not likely to contribute to the neuron specific transcripts in tumors (Supplementary Fig. S3b). Clinically, high levels of neural synaptic transcripts (SYP and SNAP25) are associated with inferior prognosis and advanced tumor stage in pan-cancer TCGA tumors, indicating a tumor promoting role of TME neural signals (Supplementary Fig. S3c, d). We applied ssGSEA^25,26^ with neuron specific gene sets to quantify the activity of neural signals in individual tumors based on the tumor transcriptome (Fig. 2b). The brain-enriched pattern of neural signals in both normal and tumor tissues is confirmed in ssGSEA-based analysis (Fig. 2b and Supplementary Fig. S3e). Notably, CRE activity (ssGSEA scores of CREB1/ATF1 target gene set) is positively correlated with neural signals in both TCGA (pan-cancer) and METABRIC (breast cancer) datasets (*r* = 0.4777 and 0.4180 for TCGA and METABRIC, respectively, Fig. 2c). CRE activity is positively correlated with neural signals in 24/32 of the tumor cohorts (METABRIC and 31 TCGA cohorts, Supplementary Fig. S3f). Moreover, positive correlation between CRE activity and stemness score is observed in 28/32 of the tumor cohorts (Supplementary Fig. S3f). These data reveal that both CRE activity and stemness function are associated with TME neural signals.

To determine the spatial association of TME neural signals and cancer-cell CRE activity, we examined the localization of neural markers and CRE activity in situ. Immunofluorescence of Neurofilament Light Chain (NF-L, neural fibers), Tyrosine Hydroxylase (TH, catecholamine nerves) and synaptophysin (SYP) confirm the presence of neural signals in both human and mouse breast tumors (Fig. 2d). Notably, proximity analyses between neural markers and nuclear phospho-CREB1/ATF1 indicate that higher cancer-cell CRE activity is present in the region proximal to neural signals (NF-L, TH and SYP positive area) in breast tumors (Fig. 2e). These data indicate a direct contact between TME neural signal and cancer-cell CRE activity, indicating that spatialized adrenergic signaling (TH positive area) may activate cancer-cell CRE activity. Chronic stress-induced adrenergic signaling is critical for the activation of CRE transcription factors.^37,40^ Reanalysis of an ovarian cancer cohort (GSE9116) confirms that chronic stress is associated with both enhanced stemness and higher CRE activity in tumors (Supplementary Fig. S4a, b). We have previously developed a chronic stress model with breast cancer xenografts.^41^ A limit-dilution assay in chronic stress model confirmed higher stem cell frequency (1/318.8 and 1/62.6 in control and stressed group, respectively) in stressed group compared with control NOD-SCID IL2Rg^null^ (NSG) mice (Fig. 2f). Indeed, both MDA-MB-231 and Py8119 tumors from stressed mice exhibit higher nuclear phospho-CREB1/ATF1 intensity compared with tumors from unstressed mice (Fig. 2g and Supplementary Fig. S4c). These findings illustrate a direct bond between TME adrenergic signals and cancer-cell CRE activity in breast cancer, indicating that TME adrenergic signals may activate CRE factors in cancer cells.

Next, we determined the TME neurotransmitters that activate CRE transcription factors in cancer cells. Both epinephrine and norepinephrine transiently activate phospho-CREB1/ATF1 in MDA-MB-231, T47D, and H460 cells, whereas glutamate and acetylcholine show less effects on phospho-CREB1/ATF1 (Fig. 2h and Supplementary Fig. S4d, e). In consistent, both epinephrine and norepinephrine enhance CRE reporter activity in MDA-MB-231 and T47D cells (Fig. 2i and Supplementary Fig. S4f). Functionally, both epinephrine and norepinephrine increase the number of sphere-forming cells in MDA-MB-231, T47D and H460 cells (Fig. 2j and Supplementary Fig. S4g–i). In contrast, in DLD1 cells that show mild phospho-CREB1/ATF1 response, norepinephrine and epinephrine exhibit mild effects in both CRE reporter and sphere-forming assays (Supplementary Fig. S4f and i). Although both norepinephrine and epinephrine activate CRE dependent cancer stemness, adrenergic nerves primarily produce norepinephrine in the TME.^42^ The kinetics of norepinephrine and epinephrine induced phospho-CREB1/ATF1 response indicate more prolonged effect on ATF1/CREB1 activation by norepinephrine (Supplementary Fig. S4j). In addition, low concentration in the serum (<100 nM)^41,43^ of both epinephrine and norepinephrine may not be sufficient to activate cancer stemness via CRE dependent program, indicating an critical role of microenvironment localized norepinephrine in activating CRE dependent cancer stemness.

To determine the dynamics of norepinephrine activity in the localized TME, we introduced MDA-MB-231 cells expressing fluorescent norepinephrine sensor GRAB_NE2h_.^44^ In vitro treatment of norepinephrine (5 μM) induced rapid increases in green fluorescent signal, but not the control tdTomato signal (Supplementary Fig. S4k–l). The MDA-MB-231 cells expressing GRAB_NE2h_, which show more sensitive response to norepinephrine, were implanted in NSG mice subcutaneously to develop a time-lapse system for imaging norepinephrine dynamics in vivo (Fig. 2k). Two-photon microscope indicated clusters of GRAB_NE2h_ active (membrane localized green fluorescent signal) cancer cells in the xenograft (Fig. 2l and Supplementary Fig. S4m). We next treated mouse with desipramine, a synaptic norepinephrine transporter (NET) blocker that suppress the recycling of norepinephrine released into the synaptic cleft. Notably, desipramine treatment induced a transient activation of GRAB_NE2h_ in the subset of cancer cells, indicating that a transient increase of localized norepinephrine in response to desipramine (Fig. 2l and Supplementary Movies S1). In addition, knocking-down the norepinephrine receptor in cancer cells with doxycycline (Dox) inducible shRNA (iDox-shADRB2) impaired the norepinephrine-induced activation of cancer stemness in sphere formation assays (Supplementary Fig. S4n–o). ADRB2 deficient MDA-MB-231 cells showed impaired stemness indicated by limit-dilution assay in NSG mice (Supplementary Fig. S4p). These data suggest that localized norepinephrine in the TME contribute to the majority of cancer-cell CRE activity in vivo. Taken together, these findings illustrate a novel cancer stemness model where cancer cell employs TME neural signal to acquire CRE driven stemness (Fig. 2m).

### ATF1 dictates adrenergic signal to potentiate stemness

**Fig. 3:**
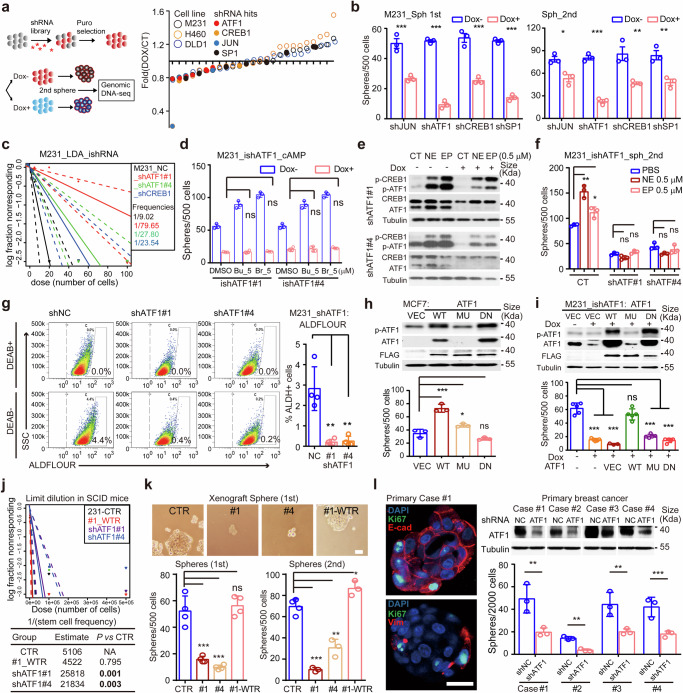
ATF1 dictates TME neural signals to potentiate cancer stemness. **a** A scheme illustrating the doxycycline (Dox) inducible shRNA (iDox-shRNA) library based functional screen to identify candidate CRE transcription factors for cancer stemness in three independent cancer cell lines (MDA-MB-231, H460 and DLD1). Top shRNA hits were labeled with distinct dots (blue, orange, black and red for oligos targeting JUN, CREB1, SP1 and ATF1, respectively). **b** Primary (left panel) and secondary (right panel) sphere formation of MDA-MB-231 cells expressing iDox-shRNAs against ATF1, CREB1, JUN and SP1. Cells were pretreated with Dox for 3 days prior to sphere formation assays (*n* = 3; mean ± SD; *P* values, Tukey’s multiple comparisons after 1-way ANOVA). **c** In vitro limit dilution assay of iDox-shATF#1/#4 and iDox-shCREB1 cells of MDA-MB-231. Differences in stem cell frequencies were determined by ELDA (https://bioinf.wehi.edu.au/software/elda/). *n* = 6 for each group. **d** Sphere formation of MDA-MB-231 iDox-shATF1 (shRNA#1 and #4) cells treated with Dox in combination with DMSO or cAMP mimics for 3 days prior to sphere formation assays (*n* = 3; mean ± SD; *P* values, Tukey’s multiple comparisons after 1-way ANOVA). **e** Western blot analysis of iDox-shATF1 MDA-MB-231 cells with/without Dox treatment with indicated antibodies. Cells were treated with epinephrine (EP, 0.5 μM) and norepinephrine (NE, 0.5 μM) for 30 min before harvesting. **f** Secondary sphere formation of iDox-shATF1 MDA-MB-231 cells. Cells with/without Dox were treated with NE/EP (0.5 μM) in sphere formation assays. **g** Flow cytometry analysis for ALDEFLUOR activity among iDox-shATF1 MDA-MB-231 cells (shRNA#1 and #4, left panel). Differences of ALDEFLUOR^+^ cells among groups were determined by one-way ANOVA (right panel). **h** Western blot analysis of MCF-7 cells expressing vector (Vec) or different forms of ATF1 (wild type (WT), S63A (MU) and R231L (DN), upper panel). Sphere formation of MCF-7 cells expressing different forms of ATF1 (lower panel). **i** Western blot analysis of iDox-shATF1#1 MDA-MB-231 cells expressing vector (Vec) or different forms of ATF1(wild type (WT), S63A (MU) and R231L (DN)). Sphere formation of iDox-shATF1#1 MDA-MB-231 cells rescued with vectors or ectopic ATF1 (lower panel). For **f**–**i**, *n* = 3; mean ± SD; *P* values, Tukey’s multiple comparisons after 1-way ANOVA. **j** In vivo limit dilution assay of iDox-shATF1 (shRNA#1 and #4) or iDox-shATF1 rescued (#1_WTR) MDA-MB-231 cells. Differences in stem cell frequencies were determined by ELDA (*n* = 10 for each group). **k** Sphere formation of MDA-MB-231 derived xenografts from **j**. Scale bars, 60 μm. **l** Left panel, immunofluorescence of E-cadherin (E-cad), Vimentin (Vim), Ki67 and DAPI in primary human breast cancer cells maintained in Matrigel. Right panel, Western blot analysis of primary breast cancer cells expressing shNC (control) or shATF1#1 vectors (pLKO.1_GFP_shRNA). Sphere formation of primary breast cancer cells from four cases (Case #1-#4) expressing shATF1 or shNC (nontarget control, (for **k**–**l**, *n* = 3; mean ± SD; *P* values, Tukey’s multiple comparisons after 1-way ANOVA). *P < 0.05; **P < 0.01; ***P < 0.001; ****P < 0.0001; ns not significant

To identify specific CRE factors required for stem-like function, we performed a functional screening in serial sphere formation with Dox inducible shRNA (iDox-shRNA) library. Among the six candidate stemness transcription factors, ATF1, CREB1, JUN, SP1 and PAX8 are associated with cAMP signaling,^45,46^ whereas BACH2 acts independent of cAMP (Fig. 3a). The shRNAs targeting ATF1 and CREB1 are consistently decreased in MDA-MB-231, NCI-H460 and DLD1 spheres, indicating impaired cancer stemness in ATF1/CREB1 deficient cells (Fig. 3a). In line with pooled screening, both primary and secondary sphere-forming capacities are impaired in cells expressing shRNA against individual cAMP activated transcription factors (ATF1, CREB1, JUN and SP1, Fig. 3b, c and Supplementary Fig. S5a). Indeed, the ATF1 depletion exhibits the most profound inhibitory effect on sphere formation (Fig. 3b, c). The levels of ATF1/CREB1 in immortalized epithelial (MCF-10A) and a panel of breast, colon and lung cancer cells indicate the presence of ATF1 in multiple cancer types (Supplementary Fig. S5b). Notably, cAMP mimics fail to potentiate the sphere-forming efficiency in ATF1 depleted MDA-MB-231 cells (Fig. 3d). ATF1 deficient cells show poor response to either epinephrine or norepinephrine, as indicated by phospho-CREB1/ATF1, CRE-dGFP reporter and sphere formation assays (Fig. 3e, f and Supplementary Fig. S5c). In contrast, both cAMP mimics and neurotransmitters partially enhance sphere formation in CREB1 depleted cells (Supplementary Fig. S5d). These data reveal a prominent role of ATF1 in neural signal-cAMP directed cancer stemness.

We next assessed the stem-like function of iDox-shATF1 cells in the Dox^+^ and Dox^-washout^ settings, where depleted ATF1 expression is recovered 4-day after the removal of Dox. Whereas Dox alone does not affect the sphere formation of parental MDA-MB-231 cells, sphere-forming frequency is recovered in the Dox^-washout^ group of iDox-shATF1 cells (Supplementary Fig. S5e). We examined whether ATF1 is required for the stemness marker defined CSC using ALDH, which has been well-established stemness marker in mesenchymal breast cancer cells.^47^ In agreement with stem-like function, ATF1 depletion results in the ablation of ALDH^+^ CSC population in MDA-MB-231 cells (Fig. 3g). The impaired sphere-forming efficiency in ATF1 deficient settings is verified in breast (SUM149, T47D), colon (DLD1) and lung cancer (A549 and H460) cells (Supplementary Fig. S5f, g), indicating ATF1 as a conserved activator of cancer stemness. Over-expression of wild-type (WT) ATF1, but not trans-activation (MU, S63A)^48^ or DNA-binding (DN, R231L)^49^ deficient mutants, enhances the sphere-forming capacity of MCF7 and BT-549 cells (Fig. 3h and Supplementary Fig. S5h). Consistently, sphere formation is recovered by WT, but not MU or DN form of ATF1 in iDox-shATF1 MDA-MB-231 cells (Fig. 3i). Over-expression of ATF1 in MCF10A-HRAS^G12V^ and HCC1937 cells increases the sphere-forming activity, confirming the role of ATF1 in potentiating stemness (Supplementary Fig. S5i). In agreement with the stem-like phenotype, the levels of pluripotent factor NANOG and MYC are decreased by shATF1 and rescued by ectopic ATF1 expression, confirming the impaired stemness in ATF1 deficient cells (Supplementary Fig. S5j). These data demonstrate a conserved, *trans*-activation dependent role of ATF1 in potentiating cancer stemness.

To confirm the function of ATF1 in vivo, we carried out a limit-dilution assay in NOD-SCID mice with MDA-MB-231 cells expressing iDox-shATF1 (#1 and #4) or iDox-shATF1#-WTR (rescued with a shRNA-resistant wild-type ATF1). Consistent with our in vitro findings, the frequencies of CSCs are decreased in ATF1 depleted groups (1/25818 and 1/21834 in shATF1#1 and shATF1#4, respectively), compared with ATF1-proficient groups (1/5106 and 1/4522 in control and shATF1#-WTR, respectively, Fig. 3j and Supplementary Fig. S5k). In contrast, modest effects on tumor growth were detected in limit-dilution assay (Supplementary Fig. S5l). Immunoblotting and immunohistochemistry (IHC) of xenografts confirm the depletion of ATF1 in shATF1 groups (Supplementary Fig. S5m, n). In addition, ATF1 deficiency impairs the sphere formation in cancer cells derived from xenografts (Fig. 3k), whereas cell proliferation is modestly affected (Supplementary Fig. S5o). Moreover, depletion of ATF1 in primary breast cancer cells confirms that ATF1 deficiency abrogates the sphere formation in all of the four primary cancer cells (Fig. 3l and Supplementary Fig. S5p–q). Collectively, the data establish ATF1 as a specific CRE transcription factor that dictates TME neural signals to enable cancer stemness.

### ATF1 *trans*-activates both pluripotent and mitochondrial genes

**Fig. 4:**
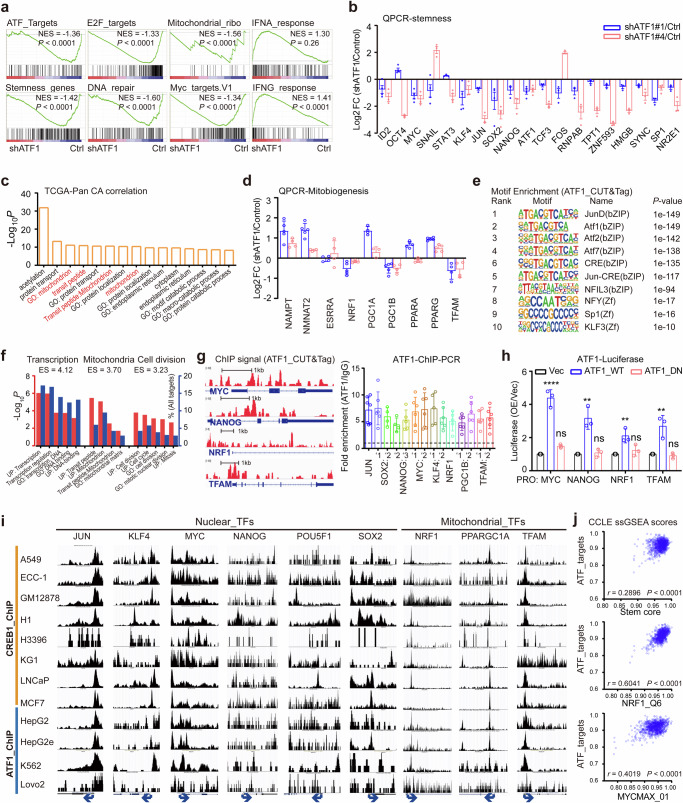
ATF1 *trans*-activates core pluripotent and mitochondrial factors. **a** Representative GSEA enrichment plots for gene sets enriched in control and shATF1 (shATF1#1 and shATF1#4) groups. **b** QRT-PCR (QPCR) analysis of stemness genes in iDox-shATF1 MDA-MB-231 cells. Fold changes (presented as Log2) in gene expression were compared to cells without Dox treatment (*n* = 3; mean ± SD). **c** Top biological processes enriched in ATF1 co-expressed genes based on the TCGA pan-cancer datasets. *P* values were EASE scores (modified Fisher Exact *P* value) provided in the DAVID database. **d** QPCR analysis of mitochondrial biogenesis genes in iDox-shATF1 MDA-MB-231 cells. Fold changes (presented as Log2) in gene expression were compared to cells without Dox treatment (*n* = 3; mean ± SD). **e** Top motifs enriched in ATF1 binding DNA element in MDA-MB-231 cells determined by de novo motif analysis of ATF1-CutTag peaks. **f** Biological processes enriched in ATF1 target genes, as determined by genes with transcription start sites 2k bp from in ATF1 binding peaks. **g** Left panel, representative tracks of normalized ATF1 CUT&TAG-seq signals at *MYC*, *NAONG*, *NRF1* and *TFAM* loci. Right panel, PCR quantification of ATF1 ChIP products in the regulatory regions of stemness and mitochondrial regulators. ATF1 ChIP signal was normalized over control IgG (*n* = 3; mean ± SD). **h** Luciferase reporter assay for ATF1 mediated gene transactivation. 293 T cells were co-transfected with a promoter-Firefly-luciferase reporter construct (MYC, NANOG, NRF1 and TFAM) in combination with empty vector, wild-type or mutant ATF1. Results were expressed as ratio of Firefly/ Renilla and normalized to vector construct (*n* = 4; mean ± SD; (*n* = 3; mean ± SD; *P* values, Tukey’s multiple comparisons after 1-way ANOVA). **i** The ENCODE ChIP-seq datasets (https://www.genome.ucsc.edu/index.html) showing ATF1 and CREB1 peaks in the regions of nuclear and mitochondrial regulators according to the data from the UCSC Genome Browser. Red boxes represent region near the transcription start site. Blue arrows represent transcription orientation. **j** Pearson correlations among ATF1, MYC, NRF1 and stemness ssGSEA scores based on the transcription profiles of the CCLE cancer cell lines (*P* values, Pearson correlation). *P < 0.05; **P < 0.01; ***P < 0.001; ****P < 0.0001; ns not significant

To decipher the transcriptional targets of ATF1, we performed RNA-sequencing in iDox-shATF1 MDA-MB-231 cells. Quantitative reverse-transcribed PCR (QPCR) validated the differentially expressed genes in RNA-seq (Supplementary Fig. S6a, b). GSEA indicates that ATF1 deficiency reduces the levels of stemness genes, ATF targets and MYC targets (Fig. 4a). Reduced nuclear pluripotent factors (including MYC, NANOG, KLF4 and SOX2) in ATF1 deficient groups were further confirmed by QPCR analysis (Fig. 4b). Notably, genes involved in mitochondrial ribosome are reduced in response to ATF1 depletion (Fig. 4a). Analysis of ATF1 co-expressed genes in pan-cancer TCGA dataset confirms that genes associated with mitochondrial function are among the top ATF1 co-expressed genes (Fig. 4c). Consistently, genes responsible for mitochondrial biogenesis (NRF1, PPARGC1B and TFAM1), mitochondrial dynamics (MFN1/2, OPA1, DRP1 and MIEF1) and mitochondrial bioenergetics (ATP5A1, COX7C and CYB5B) are down-regulated in ATF1 deficient cells (Fig. 4d and Supplementary Fig. S6c). These data indicate that both nuclear pluripotency factors and mitochondrial biogenesis regulators are transcriptional targets of ATF1.

To determine the direct binding of ATF1 to candidate transcriptional targets, we performed a Cleavage Under Targets and Tagmentation (CUT&Tag) assay.^50^ The top enriched CRE motifs confirm the specificity of ATF1 occupancy in the CUT&Tag assay (Fig. 4e). Annotation of 749/541 genes within 2000bp of ATF1-bound regions indicate that genes in cell division and mitochondrial biogenesis are top enriched (Fig. 4f). Indeed, ATF1 directly binds to the promoters of both pluripotent and mitochondrial factors (Fig. 4g and Supplementary Fig. S6d). The binding of promoters within candidate factors (MYC, NANOG, NRF1 and TFAM) were validated by PCR analysis of chromatin DNA immunoprecipitated by ATF1 (ChIP-PCR assay, Fig. 4g). To determine whether ATF1 enhances the transcription of target genes, we constructed promoter-driven luciferase reporters of both pluripotent (MYC and NANOG) and mitochondrial (NRF1 and TFAM) factors. Ectopic expression of wild-type, but not inactive ATF1 enhances the luciferase activity of luciferase reporters for MYC, NANOG, NRF1 and TFAM (Fig. 4h). Thus, ATF1 directly *trans*-activates nuclear pluripotency factors and mitochondrial biogenesis regulators.

To ask whether ATF1 binding at nuclear pluripotency factors and mitochondrial biogenesis regulators are conserved across different cells, we analyzed the ATF1/CREB1 ChIP-seq datasets from the ENCODE project. In fact, a conserved binding of ATF1/CREB1 at the promoters of nuclear pluripotent and mitochondrial biogenesis regulators are detected (4/4 cell lines for ATF1 and 8/8 cell lines for CREB1, respectively). Regions near the promoters of nuclear pluripotent factors such as MYC, NANOG, KLF4 and SOX2 exhibit ChIP peaks in both ATF1 and CREB1 ChIP datasets. Promoters of core mitochondrial biogenesis factors (NRF1 and TFAM) are also occupied by ATF1/CREB1 (Fig. 4i). Moreover, ssGSEA analysis with transcription factor gene sets in 1019 human cancer cell lines (The Cancer Cell Line Encyclopedia,^51^ CCLE dataset) indicates that CRE transcription factor (ATF1 and CREB1) activities are positively correlated with MYC targets, NRF1 targets and stemness scores (Fig. 4j and Supplementary Fig. S6e). These data uncover a conserved role of ATF1 in *trans*-activating both nuclear pluripotency and mitochondrial biogenesis across multiple cancer cells.

Transcriptomic profiling indicates increased transcription of genes involved in both cellular senescence and interferon response in ATF1 depleted cells, indicating the emergence of senescent-like phenotype after ATF1 deficiency (Fig. 4a and Supplementary Fig. S6f). In line with the transcriptional pattern, ATF1 deficient cells show deceased proliferation and increased apoptosis (Supplementary Fig. S6g–i). Notably, the presence of senescent associated β-gal (SA-β-gal) and the reduction of phosphorylated RB in shATF1 cells indicate senescent-like phenotype (Supplementary Fig. S6j, k). In ATF1 deficient cells, the proportion of SA-β-gal^+^ cell is 4-fold higher (~20% compared with 5% SA-β-gal^+^ population) in label retaining cells (slow-cycling cells that are enriched for CSCs) compared with that of total cells (Supplementary Fig. S6l). In addition, SA-β-gal staining in oncospheres, which are enriched for CSCs, show more SA-β-gal^+^ cells in ATF1 deficient cells compared with ATF1 competent cells (Supplementary Fig. S6m). The results indicate that ATF1 depletion leads to senescence-like phenotype in stem-like cancer cells.

### ATF1 deficiency impairs mitochondrial rejuvenation

**Fig. 5:**
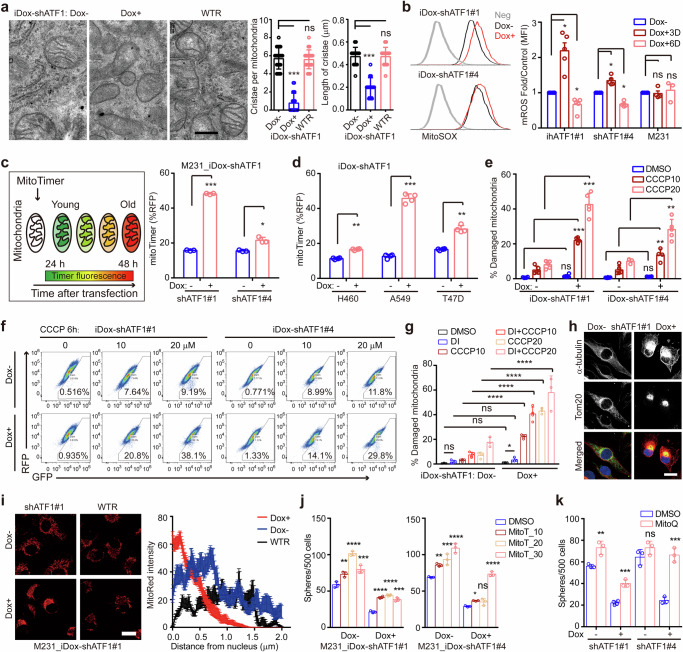
ATF1 depletion impairs mitochondrial rejuvenation. **a** Representative images of the mitochondria in iDox-shATF1 MDA-MB-231 (Dox −/+) and iDox-shATF1-ATF1 rescued cells under the electron microscope. Bar graphs show the results from the morphometric analysis of cristae number or cristae length/mitochondrial area in cells (*n* = 20 mitochondria/group; mean ± SD). Scale bar, 0.2 μm. **b** Mitochondrial ROS as indicated by flow cytometry analysis of MitoSOX Red. Parental and iDox-shATF1#1/#4 MDA-MB-231 cells were treated with Dox for 3 days or 6 days (*n* = 3; mean ± SD). **c** Mitochondrial turnover determined by flow cytometry analysis of cells 48 hr after transiently transfected with mitoTimer (left panel. *n* = 3; mean ± SD). **d** Mitochondrial turnover as determined by flow cytometry analysis of cells 48 hr after transiently transfected with mitoTimer in iDox-shATF1 H460, A549 and T47D cells (*n* = 3; mean ± SD). **e** Percentages of cells with damaged mitochondria as indicated by MitoTracker Deep-Red^low^ Green^high^ cells (*n* = 3; mean ± SD). For **a**–**e**, *P* values, Tukey’s multiple comparisons after 1-way ANOVA. **f** Representative plots showing mitochondrial damage as determined by flow cytometry analysis of MitoTracker Deep-Red and MitoTracker Green. The iDox-shATF1#1/#4 MDA-MB-231 cells were treated with Dox for 4 days, challenged with CCCP for 6 h before MitoTracker staining. **g** Percentages of cells with damaged mitochondria as indicated by MitoTracker Deep-Red^low^ Green^high^ cells treated with CCCP in combination with dynein inhibitor (DynI). *n* = 3; mean ± SD; *P* values, Tukey’s multiple comparisons after 1-way ANOVA. **h** Immunofluorescence of Tom20 and alpha tubulin in iDox-shATF1#1 MDA-MB-231 cells treated with Dox for 4 days. Scale bars, 20 μm. **i** Left panel, mitochondria localization determined by confocal microscopy using MitoTracker Red staining. Right panel, MitoTracker Red intensity (mean ± SD) as a function of distance to nuclei was analyzed (*n* = 28 for CT group, *n* = 52 for Dox group and *n* = 20 for wild-type rescued group, respectively). Scale bar, 20 μm. **j** Sphere formation of iDox-shATF1 MDA-MB-231 cells with/without Dox treatment, cells were rescued with MitoTempo (MitoT, 10, 20, 30 μM), and collected for sphere forming assays. **k** Sphere formation of iDox-shATF1 MDA-MB-231 cells with/without Dox treatment, cells were rescued with MitoQ (0.05 μM), and analyzed for sphere forming capacity (for **j**–**k**, *n* = 3; mean ± SD; *P* values, Tukey’s multiple comparisons after 1-way ANOVA). *P < 0.05; **P < 0.01; ***P < 0.001; ****P < 0.0001; ns not significant

Mitochondrial biogenesis is required for cancer stemness.^52^ To determine how mitochondrial status is controlled by ATF1, we analyzed mitochondrial activity with MitoTracker Red in iDox-shATF1 cells. Active mitochondria (indicated by MitoTracker Red intensity) in cells are gradually reduced after 6-day Dox treatment, indicating chronically impaired mitochondria after ATF1 depletion (Supplementary Fig. S7a). Transmission electron microscopy demonstrates an 80% reduction in cristae number, and a 60% decrease in cristae length per mitochondrion in shATF1 cells compared with the mitochondria in either control or wild-type ATF1 rescued cells (Fig. 5a). Transient ATF1 depletion with 3-day Dox induction increases mitochondrial reactive oxygen species (mROS, indicated by MitoSOX intensity), whereas prolonged ATF1 depletion decreases mROS in iDox-shATF1 cells (Fig. 5b and Supplementary Fig. S7b). The sequential change in mROS is consistent with the phenotype of mitochondrial dysfunction induced by impaired mitochondrial biogenesis, which generates transient oxidative stress and dysfunctional mitochondria.^53^ Dysfunctional mitochondria in ATF1 deficient cells are also supported by impaired mitochondrial membrane potential in both MDA-MB-231 and A549 (Supplementary Fig. S7c, d). Accordingly, ATF1 depletion results in dysfunctional mitochondria.

The direct *trans*-activation of mitochondrial biogenesis factors by ATF1 suggests that dysfunctional mitochondria in ATF1 depleted cells may result from impaired mitochondrial turnover, a process that rejuvenates old mitochondria with newly formed mitochondria. A mitochondria-localized Timer (mitoTimer) reporter has been developed to assess mitochondrial turnover according to the time-dependent shift from green to red fluorescence of mitoTimer.^54^ Consistent with our hypothesis, ATF1 depletion in iDox-shATF1 cells results in increased proportion of cells with low mitochondrial turnover rates in MDA-MB-231, T47D, A549 and H460 cells (Fig. 5c, d and Supplementary Fig. S7e). To gain insights into the mitochondrial dysfunction after ATF1 depletion, we examined the extent of damaged mitochondria by MitoTracker Green/Red staining^55^ in response to carbonyl cyanide m-chlorophenyl hydrazone (CCCP). The proportion of cells with damaged mitochondria (MitoTracker Green^high^/Red^low^ cells) is comparable after ATF1 depletion. However, an increase in the proportion of damaged mitochondria in the shATF1 group is present compared with those of control cells in the presence of CCCP (Fig. 5e, f). The effect of Dox on CCCP-induced mitochondrial damaged is excluded by the findings that Dox does not alter the extent of mitochondrial damage in either parental or iDox-shGFP MDA-MB-231 cells (Supplementary Fig. S7f, g). The vulnerability to CCCP challenge is further aggravated when ATF1 deficient cells are treated with either dynein inhibitor (Ciliobrevin D) or lysosome inhibitor (leupeptin), which suppresses mitochondrial turnover by blocking the removal of damaged mitochondria (Fig. 5g, Supplementary Fig. S7h, i). Thus, ATF1 depletion reduces mitochondrial rejuvenation to induce dysfunctional mitochondria.

Perinuclear mitochondria clustering is a distinct feature of cells with dysfunctional mitochondria.^56^ Immunofluorescence with Tom20 and α-tubulin antibodies indicates perinuclear clustered mitochondria in ATF1 depleted cells (Fig. 5h). Indeed, live cell imaging with MitoTracker Red confirms that ATF1 depletion either by siRNA or shATF1 results in perinuclear clustered mitochondria, which is rescued by ectopic ATF1 expression in shATF1 cells (Fig. 5i and Supplementary Fig. S7j). The localization of mitochondria is not affected by Dox alone in parental MDA-MB-231 cells (Supplementary Fig. S7k). In addition, ATF1 depletion-induced perinuclear mitochondria is confirmed in both A549 and T47D cells, indicating a conserved mechanism of ATF1-dependent mitochondrial dysfunction (Supplementary Fig. S7l, m). Restraining the mitochondrial function by either DCNT2 overexpression or dynein inhibition, which blocks the rejuvenation of functional mitochondria, impairs stem-like function in ATF1 deficient cells (Supplementary Fig. S7n). Mitochondria targeted antioxidants (MitoTempo or MitoQ) that scavenge mROS were applied to restore mitochondrial function. Indeed, both MitoTempo and MitoQ recover the perinuclear mitochondria localization in ATF1 depleted cells (Supplementary Fig. S7o, p). In agreement, cancer stemness indicated by both sphere-forming capacity and ALDH activity are partially rescued by MitoTempo and MitoQ in ATF1 deficient cells (Fig. 5j, k and Supplementary Fig. S7q, r). Collectively, these data suggest that deficient mitochondrial biogenesis induces dysfunctional mitochondria to impair cancer stemness in ATF1 depleted cells.

### Nucleus-mitochondria coordination underlies cancer stemness

**Fig. 6:**
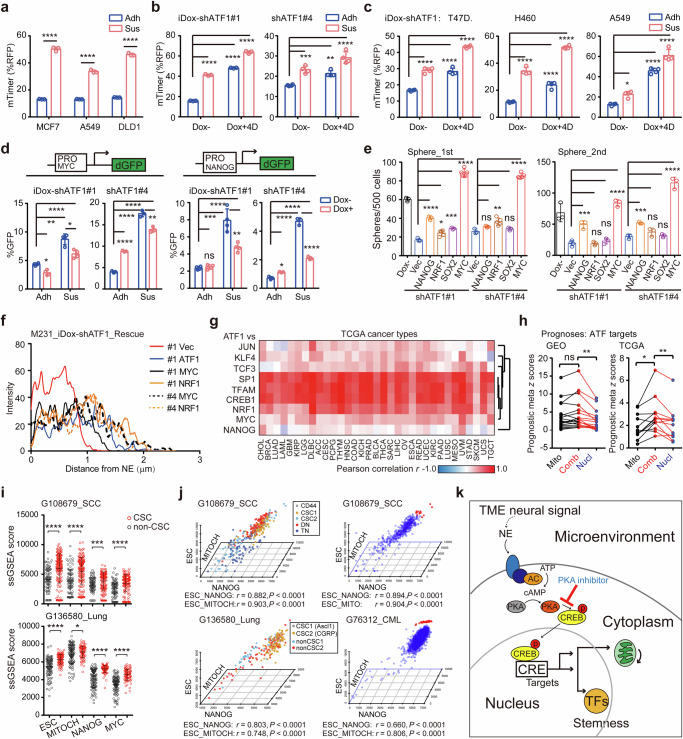
ATF1 coordinated nucleus-mitochondria state underlies cancer stemness. Mitochondrial turnover as determined by mitoTimer in MCF7, A549 and DLD1 (**a**), iDox-shATF1 MDA-MB-231 (**b**), iDox-shATF1 H460, A549 and T47D (**c**) cells. Cells in each group were transiently transfected with mitoTimer for 24 hr, seeded in adherent (Adh) or suspension (Sus) culture for 1 day and collected for flow cytometry. **d** NAONG- or MYC-Promoter activities indicated by promoter-dGFP reporter cell lines. Cells were treated with Dox for 3 days, seeded in adherent (Adh) or suspension (Sus) culture for one day followed by flow cytometry. Reporter negative cells were loaded as control. **e** Serial sphere formation of iDox-shATF1 MDA-MB-231 cells rescued with Dox inducible expression of NRF1, NANOG, SOX2 and MYC, respectively. For **a**–**e**, *n* = 3; mean ± SD; *P* values, Tukey’s multiple comparisons after 1-way ANOVA. **f** Mitochondria localization determined by MitoTracker Red. The iDox-shATF1#1 MDA-MB-231 cells rescued with NRF1, MYC and ATF1 were stained with MitoTracker for 30 min. MitoTracker Red intensity (mean) as a function of distance to nuclei was analyzed (*n* = 20 for each group). **g** Correlation of ATF1 expression with stemness/mitochondria genes in the 33 cancer types from the TCGA dataset. Correlations (Pearson *r*) between ATF1 and individual gene was determined according to their mRNA in tumors. **h** The prognostic meta-*z* sores of ATF1/CREB1 target genes (listed in Supplementary Table S2, classified as mitochondrial, nuclear pluripotent and combined targets, respectively) among GEO (*n* = 18) and TCGA (*n* = 12) cancer types. Meta-*z* scores were calculated by unweighted prognostic *z* scores of individual genes of the signature (*P* values, 2-sided *t* test for paired samples). **i** The ssGSEA scores of stemness (ESC), nuclear transcription factor (NANOG, MYC) and mitochondrial gene sets in CSCs and non-CSCs based on the single-cell transcriptome. Lung cancer (GSE136580) and skin squamous cell carcinoma (SCC) cells (GSE108679) were grouped by CSC markers (NE in GSE136580 and CD44/CD34/ITGA6 in GSE108679). *P* values, 2-sided *t* test. **j** The ssGSEA scores of stemness (ESC), nuclear transcription factor (NANOG) and mitochondrial gene sets in single cancer cells. Lung cancer (GSE136580) and SCC cells (GSE108679) were grouped by CSC markers. DN, double negative, TN, triple negative. Populations of chronic myeloid leukemia (CML, GSE76312) and SCC cells with high ESC, NANOG and mitochondrial scores were labeled in red. **k** Model of cAMP responsive program in rejuvenating mitochondria and reprogramming nucleus to potentiate stemness in cancer cells. A TME neural signal responsive nucleus-mitochondria program serves as a convergent mechanism underlying cancer stemness. *P < 0.05; **P < 0.01; ***P < 0.001; ****P < 0.0001; ns not significant

Mitochondrial dysfunction in ATF1 deficient cells suggests that cellular adaptivity may serve as a critical mechanism of ATF1 directed stemness program. To test this hypothesis, we examined the transient cellular response in serum-free suspension condition, where only CSCs are supposed to survive and expand into oncospheres.^57^ Transient exposure to suspension culture of bulk cancer cells thus mimics how differentiated cells (the majority of bulk cells) respond to suspension culture. MitoTimer assay indicates that mitochondrial turnover is decreased in 24-hour suspension compared with adherent cells in MCF7, DLD1 and A549 cells (Fig. 6a). Notably, ATF1 depletion further reduces mitochondrial turnover in 24-hour suspension cells in MDA-MB-231, T47D, H460 and A549 cells (Fig. 6b, c). In line with mitoTimer assay, 24-hour suspension cells show reduced mitochondrial membrane potential, which is further impaired in ATF1 deficient cells (Supplementary Fig. S8a, b). Using NANOG/MYC-promoter driven destabilized GFP (ProNANOG/MYC_dGFP) reporters, we assessed how nuclear pluripotent activity response to transient suspension culture. Transient exposure to suspension enhances NANOG/MYC-promoter activities compared with adherent cells (Fig. 6d). However, the transient increase in NANOG/MYC-promoter activities is impaired in ATF1 deficient context, indicating that ATF1 is required for the adaptive enhancement of nuclear pluripotency factors in suspension condition (Fig. 6d and Supplementary Fig. S8c). These findings indicate that ATF1 directed nuclear pluripotency and mitochondrial biogenesis enables the adaptive response to suspension condition to acquire stem-like property.

We next determined how nuclear pluripotency and mitochondrial biogenesis contribute to ATF1 mediated cancer stemness. Restoration of NANOG, SOX2 or NRF1 partially rescues the sphere formation capacity of iDox-shATF1 MDA-MB-231 cells. Indeed, re-expressing MYC restores the stem-like function of ATF1 deficient MDA-MB-231 cells (Fig. 6e and Supplementary Fig. S8d). The effects of NANOG, NRF1 and MYC on sphere formation in ATF1 deficient cells are confirmed in A549 and H460 cells (Supplementary Fig. S8e, f). Both mitochondrial and pluripotent factors (NRF1, MYC, SOX2 and NANOG) are restored by MYC, but not NANOG or SOX2 in ATF1 depleted MDA-MB-231 cells (Supplementary Fig. S8g). Quantification of a mitochondrial gene panel confirmed that MYC restores the majority of mitochondrial genes in ATF1 deficient MDA-MB-231 and A549 cells, indicating that coordinated nuclear pluripotency and mitochondrial biogenesis are required for ATF1 dependent cancer stemness (Supplementary Fig. S8h). In accordance, mitochondrial ROS in ATF1 deficient cells is rescued by NRF1, and partially rescued by MYC and NANOG (Supplementary Fig. S8i). The perinuclear mitochondria are recovered by either NRF1 or MYC in ATF1 depleted MDA-MB-231cells (Fig. 6f and Supplementary Fig. S8j). In addition, mitochondrial membrane potential is partially rescued by NANOG, MYC or NRF1 in ATF1 deficient MDA-MB-231 cells (Supplementary Fig. S8k). The recovery of mitochondrial membrane potential by both MYC and NRF1 is further validated in ATF1 depleted A549 and H460 cells (Supplementary Fig. S8l). To examine the interactions between nuclear and mitochondrial regulators, we knocked-down NRF1 or NANOG in MDA-MB-231 cells, respectively. Deficiency of either NRF1 or NANOG impairs sphere formation in MDA-MB-231 cells (Supplementary Fig. S8m). Although MYC, NANOG, CREB1 and ATF1 are not affected by shNRF1, both mitochondrial functions are partially impaired in NANOG deficient cells, indicating that mitochondrial biogenesis may serve as downstream effector of nuclear pluripotency factors such as NANOG and MYC in MDA-MB-231 cells (Supplementary Fig. S8n, o). These data reveal that coordinated nuclear pluripotency and mitochondrial biogenesis underlies ATF1 dependent cancer stemness.

To determine the clinical relevance of ATF1 in coordinating nuclear pluripotent and mitochondrial regulators in cancer, we evaluated the correlations between ATF1 and candidate genes in the TCGA dataset. In accordance, ATF1 is positively correlated with both pluripotent (33/33, 23/33 of cancer types for MYC and NANOG, respectively) and mitochondrial regulators (33/33 of cancer types for both TFAM and NRF1) in TCGA cohorts (Fig. 6g). Combined mitochondrial and nuclear targets of ATF1/CREB1 is associated with worse prognosis in cancer patients from the TCGA and GEO datasets compared with either mitochondrial or nuclear subset of targets (Fig. 6h). Survival analyses in individual cancer cohorts verify the poor prognosis in patients co-expressing nuclear and mitochondrial targets of ATF1/CREB1, confirming the clinical relevance of ATF1 coordinated nucleus-mitochondria program (Supplementary Fig. S8p, q).

To decipher the coordinated nuclear pluripotency and mitochondrial biogenesis in individual CSCs, we assessed single-cell nuclear pluripotency and mitochondrial biogenesis levels using single-cell transcriptome datasets. Both pluripotency (NANOG and MYC) and mitochondrial activities are positively correlated with stemness scores in single cancer cells (Supplementary Fig. S9a). The activities (ssGSEA scores) of both pluripotency factors (NANOG, MYC) and mitochondrial biogenesis are higher in CSCs compared with non-CSCs in skin squamous cell carcinoma (SCC) and lung cancer (GSE108679 and GSE136580, respectively, Fig. 6i). Moreover, individual CSCs in SCC (CD44^+^CD34^+^ITGA6^+^), lung cancer (ASCL1^+^/CGRP^+^ neuroendocrine cell) and breast cancer (CD44^+^CD24^-^, GSE124887) show concurrent activation of stemness genes, nuclear factors and mitochondrial components (Fig. 6j and Supplementary Fig. S9b, c). The coordinated levels of mitochondrial and pluripotent factors in single cells are confirmed in chronic myeloid leukemia (CML, GSE76312) and acute myeloid leukemia (AML, GSE140896) datasets (Fig. 6j and Supplementary Fig. S9c, d). Distinct clusters of cells with concurrent higher mitochondrial components, nuclear pluripotent factors and stemness scores are identified in SCC, CML, and AML cells. In fact, the correlations among mitochondrial biogenesis, nuclear pluripotency and stemness scores are observed both in normal and cancer cells in glioma, CML and breast cancer (Supplementary Fig. S9e). Higher mitochondrial biogenesis levels are consistently observed in cancer (stem) versus normal (stem) cells in glioma, CML, and breast tissues, respectively (Supplementary Fig. S9f, g). Correlations between StemnessScores and the ssGSEA scores of both mitochondrial and pluripotent gene sets in TCGA tumors confirm that cancer stemness is positively correlated with both pluripotent and mitochondrial factors in human cancers (Supplementary Fig. S9h). In summary, coupled activation of nuclear pluripotency and mitochondrial biogenesis, directed by TME neural signal-ATF1 pathway serves as a conserved mechanism underlying cancer stem-like state (Fig. 6k).

## Discussion

In summary, our study uncovers a conserved, cancer cell intrinsic cAMP responsive program that is specialized in employing TME neural signals to potentiate cancer stemness. The novelty includes at least four aspects. Firstly, we focus on the essential role of localized neural-cancer crosstalk as a novel mechanism of cancer stemness through hijacking microenvironmental neural signals. Secondly, we develop an unbiased screening approach that characterizes candidate TME responsive cancer stemness programs, revealing a previously unrecognized role of cAMP signaling as a specialized mode of sustaining cancer stemness in vivo. Thirdly, we have characterized ATF1 as a core CRE transcription factor mediating the crosstalk between TME neural signaling and cancer stemness. And finally, comprehensive mechanistic insights of cAMP activated cancer stemness reveals that TME neural signal driven pluripotent transcription and mitochondrial biogenesis provides coordinated transcriptional and metabolic signals for cancer stemness.

Deciphering the conserved tumor microenvironmental network that initiate stem-like state is essential for understanding and targeting CSCs. Increasing epidemiologic and biological evidence indicates that neural signals contribute to progressive disease and poor outcomes in cancer.^7,8,14^ Neurotransmitters such as norepinephrine^10^ and glutamate,^13^ are recently recognized to enhance stem-like properties. However, the direct effects of tumor infiltrated neural signals on cancer cells remains poorly understood. This study highlights CRE as a conserved sensor of cancer associated neural signals to direct cancer stemness. The localized cancer associated norepinephrine identified here is consistent with the finding that local norepinephrine accumulation promotes pancreatic cancer.^10^ Given the neuron-like behavior of cancer cells in certain context, the evaluation of neural signals using bulk RNA-seq profiles may not exclude the possibility that neuron-specific transcripts derived from cancer cells. Further studies with spatial transcriptomes and serial immunostaining in intact tissue could provide more insights into the cancer associated nerves.

The reciprocal communications between cancer cells and infiltrating nerves in the localized tumor microenvironment raise interesting issues that remains largely unexplored. On the one hand, cancer cells could actively guide the distribution of neural signals in the TME. The distribution of neurons in the tumor microenvironment is affected by tumor secreted signals such as nerve growth factor (NGF) and Brain-derived neurotrophic factor (BDNF).^8,58^ Interestingly, both NGF and BDNF are activated by CRE signaling through the cis-regulatory motifs.^59,60^ Therefore, CRE activation in cancer cells might activate neural associated factors that promote neurogenesis, thereby guiding the distribution of neurons to hijack the cellular reprogramming mechanism by which nervous system assisting tissue development and regeneration. Increasing evidence has point out that neural signals are required for the development and regeneration of tissues by interacting with stem/progenitor cells in tissues including skin, kidney, and bone marrow.^61–63^ Activation of adrenergic signaling leads to “unhealing wounds” resembling the tumor microenvironment.^64^ Thus, cancer cells could construct the innervation patterns through secreted factors (e.g. NGF and BDNF) to drive a self-assembled neural-cancer circuits that favors cancer stemness.

The wide distribution of cancer associated nerves and their potential interactions with cancer cells provide novel insights into the tumor microenvironment, offering potential vulnerabilities of cancer stemness. In the current study, several neural signal-induced effectors are potential targets for the cancer stem-like state. For instance, psychological interventions that relive chronic stress might be used to reduce the initiating signals of cAMP-CRE response cascade.^7^ Clinically approved adrenergic receptor blockers such as beta-adrenergic blocking agents might be applied to block the adrenergic receptor induced cAMP activation to suppress stemness.^10^ In addition, the critical roles of PKA in the process provide a potential application of PKA kinase inhibitors in cancer patients. Indeed, the role of CRE program in cancer stemness is consistent with a recent proteomic study that PKA activation maintains small cell lung CSCs.^65^ Our study focuses on the cancer associated nerve responsive mechanisms of CSCs and provides promising candidates for targeting cancer stemness.

Transcription factors are well accepted to control cell fate in development, regeneration and tumorigenesis. A number of master transcription factors in normal stem cells, including MYC,^3^ NANOG,^28^ JUN,^66^ and SOX2,^28^ have been identified to enhance stem-like properties in specific cancer types. However, less is known about whether cancer type specific signaling-induced transcription factors enhance stemness in different cancer types. Actually, transcription factors that enhance stemness in one context might repress stemness in another cancer type. For example, induction of the master stemness factor MYC suppresses the clonogenic potential mammary epithelial and breast cancer cells.^67^ Our data support the cancer type-specific pattern of stemness transcription factors, and identify cancer associated neural signals responsive CRE factors that convergently activate stemness program in a variety of cancer types. The findings indicate that CRE transcription factors may serve as convergent stemness drivers that link cancer associated neural signals to stem-like state.

Mitochondrial status, including mitochondrial ROS, bioenergetics and morphological dynamics has been linked to stemness.^52,68^ For instance, cells inherited with young mitochondria are stem-like, whereas cells inherited with old mitochondria are differentiated in asymmetric cell division.^68^ Although nuclear pluripotency factors and mitochondrial dynamics are long been considered as essential components of CSCs, little evidence shows how nuclear pluripotency and mitochondrial status are coordinated to enable cancer stemness in specific settings. Our results show that upon cAMP induced activation, CRE transcription factors orchestrate a coordinated nuclear-mitochondrial program, thereby driving cancer stemness. The concept is consistent with the finding that a balanced MYC and mitochondrial regulator PGC1A determines pancreatic cancer stem cells.^5^ Therefore, the coordinated nuclear pluripotency and mitochondrial biogenesis is a distinct feature of CSCs.

In summary, this study reveals a novel mechanism of cancer stemness triggered by the neural signal-ATF1 dependent coordination of nuclear pluripotency and mitochondrial biogenesis. We illustrate a new situation where cancer cell employs a TME neural signal to activate cAMP-ATF1 dependent transcriptional targets, thereby coordinating nuclear pluripotency and mitochondrial biogenesis to acquire cancer stem-like state. Our study raises several interesting questions about TME neural signal and cancer stemness that warrant further exploration, including the identity of distinct neural signals directing stemness and the mechanism of reprograming cancer associated nerves. Furthermore, deciphering TME neural signal in specific psychological contexts would provide important new insights into the intercellular control of stemness signaling and may provide novel opportunities for intervention.

## Materials and methods

### Datasets

Paired gene expression datasets for sphere-adherent cells were downloaded from the Gene Expression Omnibus (GEO, RRID: SCR_005012). Processed microarray or RNA-seq datasets were prepared for further analyses. Microarray datasets for the GTEX (RRID:SCR_013042) and 1019 cancer cell lines were downloaded from the GTEX project^69^ and Cancer Cell Line Encyclopedia (CCLE) project,^51^ respectively. The stem gene signatures were downloaded from the published data by Malta et al.^24^ The prognostic *z*-scores for individual genes were downloaded from the PRECOG^36^ dataset. Gene sets for further analyses were downloaded from the MSigBD database (RRID:SCR_016863). The gene lists for PRECOG analyses were listed in Supplementary Table S2.

### Gene set enrichment analysis

Gene set enrichment analysis (GSEA, RRID:SCR_003199)^29^ were performed in the GSEA v2.2 software. For sphere vs adherent transcriptional datasets, enrichment of transcription factor gene sets (MSigBD, C3 TF, V6.2) in sphere and adherent phenotypes were identified in each dataset. The lists of enriched transcription factors in each dataset were used to identify candidate stemness transcription factors that were frequently enriched transcription factors among datasets. For RNA-seq profiles from inducible shATF1/shCREB1 cells, GSEA was used to compare gene sets in MSigDB (V6.2). Significantly enriched gene sets in GSEA were defined using an FDR *q*-value < 0.25 and a nominal *P* value < 0.05. Single sample GSEA (ssGSEA, V10.0.1) was performed in the GenePattern server^70^ as described previously.^71^ ssGSEA enrichment score of cell marker gene sets, transcription factor target gene sets were analyzed to evaluate the activities of candidate cell markers and transcription factors, respectively. Deconvolution of cell component from bulk RNA-seq were performed based on the ssGSEA scores of each cell marker gene set in individual tumor samples. Enrichments of stemness factors in TCGA tumors were analyzed by investigating gene lists that are highly expressed in the StemnessScore^high^ tumors in the MSigDB database (cutoff values, *P* < 0.01 and False Discovery Rate (FDR) *q*-value < 0.05).

### Prognosis analysis

The prognostic scores were either downloaded from the PreCOG or generated using TCGA tumor datasets as previously described.^71^ Unweighted meta-z-scores were collapsed into a global meta-*z*-score for each gene using a Stouffer’s method (unweighted). The meta-*z*-score = (${\sum }_{i=1}^{k}{Zi}$)/$\sqrt[2]{N}$, where N represents the number of genes in the gene set. To assess the prognostic values of gene signatures, Kaplan Meier analysis of single genes or gene sets was performed in the Kaplan Meier plotter online database (http://kmplot.com/analysis/index.php?p=background) (Gyorffy et al., 2012). User selected probe set was selected, and patients were split according to the optimized cut-offs. Relapse- free survival of breast cancer, overall survival of lung, gastric and ovarian cancer was evaluated in all patients available in the database.

### Cell culture

SUM149 (RRID: CVCL_3422) cell lines were obtained from Prof. Zhiming Shao from the Fudan University Shanghai Cancer Center and maintained with F12 medium with 5% FBS, Insulin (5 μg/ml) and Hydrocortisone (1 μg/ml). Other cell lines, including MDA-MB-231 (RRID: CVCL_0062), T47D (RRID: CVCL_0553), MCF7 (RRID: CVCL_0031), BT-549 (RRID: CVCL_1092), DLD1 (RRID: CVCL_0248), A549 (RRID: CVCL_0023) and H460 (RRID: CVCL_0459) were obtained from ATCC. All cell lines were regularly tested for mycoplasma contamination using the Mycoprobe mycoplasma detection kit (R and D Systems, Minneapolis, MN). MCF-10A (RRID: CVCL_0598) cells were maintained with DMEM/F12 medium with 5% horse serum, Insulin (10 μg/ml), EGF ((PeproTech, Cat# AF-100-15, 20 ng/ml), Cholera Toxin (100 ng/ml) and Hydrocortisone (0.5 μg/ml). The other cells were maintained with growth media (DMEM for MDA-MB-231, MCF7, DLD1 and A549, and RPMI 1640 for BT-549, H460 and T47D) cultured in Heracell (Thermofisher, Waltham, MA) humidified incubators at 37 °C and 5% CO2.

### Primary breast cancer

The study of human primary tissues was approved by the ethical committee of Sun Yat-Sen University Cancer Center (#GZR2020-059). Primary tissues were obtained from Surgical department of Sun Yat-sen University Cancer Center. Tissues were processed according to the published approach. Specifically, tissues from surgical procedures were transferred to a tube containing growth media with penicillin/streptomycin (100 U/ml), and transport to laboratory at 4 °C. Tissues were cut into smaller pieces of ~3-4 mm with scissors and scalpels and transferred to 15 ml tubes. Tissues were digested at 37 °C for 6-8 hr in a DMEM media with 10% FBS, HEPES (pH7.4, 20 mM), collagenase III (1 μg/ml), hyaluronidase (1 μg/ml) and penicillin/streptomycin (100 U/ml). Epithelial cells were collected by centrifuging and filtering through 70 μm strainer. Epithelial cells were maintained in M199/F12 media with 2% FBS, Insulin (20 μg/ml), EGF (20 ng/ml), Cholera Toxin (100 ng/ml), Hydrocortisone (1 μg/ml) and penicillin/streptomycin (100 U/ml).

### Plasmid constructs

The lentiviral GFP reporter construct was constructed by modifying the lentiviral vector pLVX-AcGFP-C1 (Takara, Cat# 632155). Briefly, a mini-CMV sequence was used to replace the original elements. A degron was fused to the N-terminal of GFP to facilitate rapid response to transcriptional variations. Transcriptional reporters were constructed by inserting cis-regulatory sequences upstream of the mini-CMV. The lentiviral shRNA and doxycycline inducible shRNA plasmids were constructed based on the pLKO.1 (Addgene, Cat# 8453, RRID: Addgene_8453) and Tet-pLKO-puro (Addgene, Cat# 21915, RRID: Addgene_21915) vectors from Addgene. The pLKO.1-GFP vector was constructed by replacing the PuroR with GFP sequence in pLKO.1. The shRNA sequences targeting candidate genes were selected according to the RNAi Consortium collection (MISSION shRNA, Sigma). Oligos were synthesized and insert into the vectors according to the manual by Dr. Wiederschain. The shRNA oligos were listed in Supplementary Table S4a. Genes for over-expression were constructed by inserting cDNA of candidate genes into lentiviral vectors, including pCDH-CMV-MCS-EF1-copGFP (SBI, Cat# CD511B-1) and pLVX-TRE3G (Addgene, Cat# 6113, RRID: Addgene_6113). Norepinephrine reporter plasmid was constructed by inserting GRAB-NE2h or NE2m (amplified from the pAAV-hsyn-GRAB-NE2h/NE2m plasmid from the Li Lab) sequence into the pGF-CMV-tdTomato-LUC vector to generate the lentiviral pGF-CMV-tdTomato-T2A-NE2h/NE2m vector. Doxycycline inducible constructs were used in combination with pLVX-Tet3G (Addgene, Cat# 6111, RRID: Addgene_6111). The pMitotimer mitochondrial turnover reporter plasmid was purchased from Addgene (Cat# 52659, RRID: Addgene_52659).^54^ Lentivirus production in 293 T cells (KCB Cat# KCB 200744YJ, RRID: CVCL_0063) was performed as described previously.^71^

### Sphere formation

Adherent cells were collected by trypsin, resuspended into single-cell suspension, and seeded at 2000 cells/ml in ultra-low attachment plates (Corning, Cat# 3473). Spheres-forming medium was composed of serum-free DMEM/F12 medium containing 10 ng/ml EGF (Peprotech, Cat# AF-100-15), 10 ng/ml bFGF (Peprotech, Cat# 100-18B), 0.5 U/mL heparin (Sigma, Cat# H3149-500KU-9) and 2% B-27 (Invitrogen, Cat# 17504044). For second-generation sphere cultures, primary spheres were dissociated into single cells, and replated in sphere culture. Fresh media were added every 2 days during the sphere culture. Spheres (>60 μm in size) were counted at 7-10 days after seeding. For in vitro limit dilution assays, cells were seeded in serial doses in spheres-forming medium (100, 50, 25, 12, 6, 3, 1) in ultra-low attachment plates. Frequency was calculated using the Extreme Limiting Dilution Analysis (ELDA) ‘limdil’ function (http://bioinf.wehi.edu.au/software/elda/index.html). To screen candidate transcription factors using sphere formation, MDA-MB-231 cells were transfected with pooled shRNAs against 6 transcription factors (control shRNA (shGFP), 4 individual shRNAs for each transcription factors (ATF1, CREB1, JUN, SP1, PAX8 and BACH2, respectively)). Puromycin (Thermo, Cat # A1113802) selected cells were seeded in sphere formation culture with/without Dox for 6 days and collected for secondary sphere formation. Secondary spheres were collected for genomic DNA extraction, library construction and deep sequencing.

### Flow cytometry

Cells were collected by trypsin, resuspended into single cell suspensions. For live cells detection of fluorescent proteins, cells were washed with PBS, resuspended in PBS for analysis in Gallios cytometer (Beckman-Coulter). For cell staining, cells were washed with PBS before staining with fluorescent probes (Mitotracker Red (Thermo, Cat# M22425) /Mitotracker Green (Thermo Cat# M7514), Rhodamine 123 (Thermo, Cat# D632), MitoSOX (Thermo, Cat# M36008)) or antibodies (anti-human CD49f-FITC (BioLegend Cat# #313624, RRID: AB_2562244), anti-human EpCAM-PerCP (BioLegend Cat# 324214, RRID: AB_2098808)), washed with ice cold PBS, resuspended in PBS and load for analysis. The apoptotic cell assay was performed by the PI-Annexin V kit according to the manufacturer’s instructions. The ALDEFLUOR™ (Stemcell, Cat# 01700) analysis was performed according to the manufacturer’s protocol. Briefly, single cell suspensions were counted, washed with PBS, stained with substrate solutions with/without DEAB. ALDEFLUOR stained samples were washed, resuspended with staining buffer and load for analysis. FACS was performed in the MoFlo XDP sorter, single cells suspensions were loaded to the MDX sorter, cells were gated and sorted according to the manufacturer’s instructions.

### Western blotting

Cells or tissues were lysed using RIPA (20 mM Tris pH 7.4; 150 mM NaCl, 1% Triton X-100, 0.5% sodium deoxycholate, 0.1% SDS, 5 mM EDTA, 50 mM NaF, 0.5 mM Na3VO4, plus freshly added protease inhibitor cocktail). Protein quantification of lysates was performed using Coomassie brilliant blue G-250 as described previously. Lysates were prepared and electrophoresed using 10% SDS-polyacrylamide gels, and transferred to Immobilon-P PVDF membranes (Millipore). PVDF membranes were blocked with 5% BSA/TBST for 1 hr at room temperature prior to overnight incubation with indicated antibodies at 4 °C. Western blots were probed with the following antibodies: total α-ATF1/CREB1 (1:1000, Santa Cruz, Cat# sc-270, RRID: AB_2290030), α-phospho-CREB1/ATF1 (1:5000, ser63-ATF1/ser133-CREB1, Abcam, Cat# ab76085, RRID: AB_1523174); α-SP1 (1:1000, Santa Cruz, Cat# sc-59, RRID: AB_2171050), α-JUN (1:1000, Santa Cruz, Cat# sc-1694, RRID: AB_631263), α-LC3B (1:2000, Santa Cruz, Cat# sc-398822, RRID: AB_2877091), α-NANOG (1:2000, Abcam, Cat# ab109250, RRID: AB_10863442), α-MYC (1:2000, Proteintech, Cat# 10828-1-AP, RRID: AB_2148585), α-Flag tag (1:5000, Sigma-Aldrich, Cat# F1804, RRID: AB_262044). Antibodies to GAPDH (1:5000, Santa Cruz, Cat# sc-32233, RRID: AB_627679) and α-Tubulin (1:5000, Santa Cruz, Cat# sc-32293, RRID: AB_628412) were used as controls. Proteins were visualized with ECL (Amersham) according to manufacturer’s protocols.

### Mice models

Animal care and experimental procedures were approved by the ethical committee of Sun Yat-Sen University Cancer Center (#L025501202203014). Mice were purchased from Vital River. Psychologically-induced stress models were performed as described in our previous study.^41^ Briefly, stressed mice were restrained in a confined space to prevent them from moving freely. Breast cancer cells were injected subcutaneously into the pretreated nude mice (MDA-MB-231, 1 × 10^6^ cells) or C57/B6 mice (Py8119, 1 × 10^5^ cells) and randomly assigned them to control conditions or daily restraint stress for a maximum length of 30 days. In limit dilution studies, NOD-SCID or NOD-SCID-IL2Rg^null^ (NSG) mice were randomly grouped, and were treated with Doxycycline (2 mg/mL in drinking water with 2% sucrose) or vehicle 2 days prior to injection. Adherent cells were collected by trypsin, counted and prepared in 50% Matrigel/PBS solution at 500000, 100000, 10000, 1000/mL concentrations. Cells were kept on ice and injected subcutaneously into NOD-SCID mice (100 μL/injection). Doxycycline and vehicle control in drinking water were changed every other day, and tumor formation was monitored. Animal health status and body weight was measured every other day during the treatment. Mice were sacrificed four weeks after injection. The frequency of stem-like cells was calculated using the Extreme Limiting Dilution Analysis (ELDA) ‘limdil’ function (http://bioinf.wehi.edu.au/software/elda/index.html).

### GRAB_NE_ sensors in cultured cells and in vivo

The tdTomato positive GRAB_NE_ sensor MDA-MB-231 cells were sorted for both in vitro and in vivo studies. For in vitro imaging, cells were plated in 3.5-cm glass bottom dishes, stimulated with norepinephrine (5 μM) and imaged using ECLIPSE Ti2 (Nikon). For in vivo expression, GRAB_NE_ sensor MDA-MB-231 cells (5 × 10^5^) were implanted into the NSG mice. Tumor bearing (3-week after cell implanting) mice under isoflurane anesthesia were placed in a stereotaxic frame for Intraperitoneal injection injection of desipramine (10 mg/kg). Imaging was performed using the FVMPE-RS multiphoton microscope (Olympus) equipped with a 920-nm laser at a power setting of 1.87 W and a 1080-nm laser at a power setting of 0.80 W. Twenty images were taken at an interval of 1 minute. The fluorescent intensities in single cells were quantified using CellProfiler (version 4.2.1) using “MeasureObjectIntensity” module. CellProfiler pipelines will be provided upon request.

### SA-β-Gal staining

SA-β-Gal staining was performed by using Senescence β-Galactosidase Staining Kit (Beyotime, Cat# C0602). Cells were washed with PBS, stained with SA-β-Gal solution, and imaged according to the manufacturer’s directions.

### Colony formation analysis

Cells were seeded into 6-well plates at 250 cells/ml, incubated for 14–16 days. Growth media with/without 200 ng/ml Doxycycline were refreshed every 3 days. Colonies were stained with crystal violet and counted.

### Gene expression profiling

Total RNA was extracted using TRIzol (Invitrogen, Cat# 15596026) according to the manufacturer’s protocol. RNA samples were detected, processed and sequenced by Novogene. Briefly, cDNA libraries of indicated samples were sequenced by Illumina Hiseq 2500. Raw data were processed and analyzed using RNACocktail to generate annotated gene reads in each sample. Lists of differentially expressed genes were provided in Supplementary Table S4.

### Reverse-transcription q-PCR

Total RNA was extracted using TRIzol (Invitrogen) according to the manufacturer’s protocol, followed by cDNA generation with EasyScript One-Step gDNA Removal and cDNA Synthesis SuperMix (Transgen, Cat# AT301-02). Real-time RT-PCR was performed using SYBR Select Master Mix (Invitrogen, Cat# 4472908) in the BioRad CFX Real-Time PCR System, as recommended by the manufacturer. GAPDH was used as an internal control. The primers were listed in Supplementary Table S4b.

### Chromatin immunoprecipitation (ChIP)

Chromatin immunoprecipitation (ChIP) was performed using ChIP-IT Express Chromatin Immunoprecipitation Kits (Active Motif). MDA-MB-231 cells were crosslinked with 1% formaldehyde for 15 min at RT and then quenched with 10 ml 200 mM Glycine. The cross-linked chromatin was sheared using Covaris S220 (Covaris) following the manufacturer’s manual. Pre-cleared DNA was then used for immunoprecipitation with 10 μl α-ATF1/CREB1 (Santa Cruz, Cat# sc-270, RRID: AB_2290030) antibody or control IgG and Protein G magnetic beads at 4 °C overnight. The immune complexes were washed, eluted and reverse cross-linked according to the manufacturer’s protocol. For ChIP-PCR, DNA was extracted by phenol and phenol/chloroform extractions. The primers for ChIP-PCR analysis were listed in Supplementary Table S4c.

### CUT&Tag sequencing

CUT&Tag was performed using NovoNGS® CUT&Tag 2.0 High-Sensitivity Kit (NovoProtein, Cat #N259-YH01, RRID:AB_2886259) and optimized as previously described.^50^ Briefly, cells were harvested, counted and washed twice in 1.5 mL Wash Buffer (20 mM HEPES pH 7.5; 150 mM NaCl; 0.5 mM Spermidine; 1× Protease inhibitor cocktail). Cells were mixed with activated Concanavalin A beads and magnetized to remove the liquid with a pipettor and resuspended in Wash buffer (20 mM HEPES pH 7.5, 150 mM NaCl, 0.5 mM spermidine and Roche EDTA-free protease inhibitor). After successive incubations with primary antibody (α-ATF1/CREB1, Santa Cruz, Cat# sc-270, RRID: AB_2290030, 4 °C, 16 hr) and secondary antibody (RT, 1 hr) in Wash buffer, the beads were washed and resuspended in pAG-Tn5 at 12.5 nM in 300-Wash buffer (Wash buffer containing 300 mM NaCl) for 1 hr. Tagmentation was performed for 1 hr in 300-Wash buffer supplemented with 10 mM MgCl_2_ in a 50 µL volume. Tagmentation was stopped and the chromatin complex was digested with 10 µL 0.5 M EDTA, 3 µL 10% SDS and 2.5 µL 20 mg/mL Proteinase K at 55 °C for 1 hr. The transposed DNA fragments was purified using a Qiagen MinElute PCR Purification Kit and amplified using NEBNext Ultra II Q5 Master Mix (New England Biolabs, Cat# M0544L) with index primers. The barcoded libraries were mixed to achieve equimolar representation as desired aiming for a final concentration as recommended by the manufacturer for sequencing by Novogene using Illumina HiSeq 2500.

The raw 150 bp paired-end sequencing reads were filtered out using the trim_galore and were then aligned to the reference human genome (GRCh37/hg19) using HISAT2^72^ with X, no-spliced-alignment, and no-temp-splicesite parameters. PCR duplicates were removed using picard MarkDuplicates.^73^ Peaks were called using MACS2^74^ and annotated using HOMER.^75^

### Luciferase reporter assay

Cells were plated at a density of 2 × 10^5^ cells/well in 12-well plates. After 24 hr, cells were co-transfected with renilla luciferase (Rluc) control plasmids (pRL-TK, Addgene, Cat# 3950) and luciferase constructs (pGL4-reporter, Cat# 48744, RRID: RRID: Addgene_48744). Fluc/Rluc activities were measured using the Dual Luciferase Reporter Assay System (Promega, Cat# E1910).

### RNA interference

The siRNAs were purchased from GenePharma. siRNAs were transfected following the manufacturer’s instructions. The target sequences of siRNAs were listed in Supplementary Table S4a.

### Live cell and immunofluorescence analyses

For live cell imaging of mitochondrial dynamics, cells were stained with MitoTracker Red for 30 min in Phenol red-free medium. In immunofluorescence, cells or tissues were fixed with 4% paraformaldehyde and permeabilized with 0.5% Triton X-100/PBS. Cells were incubated with indicated primary antibodies (α-ATF1/CREB1, 1:200, Santa Cruz, Cat# sc-270, RRID: AB_2290030; α-phospho-CREB1/ATF1, 1:2000, Abcam, Cat# ab76085, RRID: AB_1523174; α-NF-L, 1:200, Cell Signaling Technology Cat# 2837, RRID: AB_823575; α-TH, 1:200, Novus Cat# NB300-109, RRID:AB_10077691; α-SYP, 1:200, Novus Cat# NBP2-25170, RRID:AB_2814699; α-Tom20, 1:200, Proteintech, Cat# 11802-1-AP, RRID: AB_2207530, 1:200; α-Ac-α-tubulin, 1:200, Cat# 32-2700, RRID: AB_2533073, 1:200) overnight at 4°C. Next, the cells were exposed to secondary antibodies conjugated to Alexa-488 Goat anti-Rabbit IgG (Invitrogen, Cat# A-11034, RRID: AB_2576217), Alexa-488 Donkey anti-mouse IgG (Invitrogen, Cat# R37114, RRID: AB_2556542) or Goat anti-Mouse IgG Alexa-546 (Invitrogen, Cat# A-21123, RRID: AB_2535765), Goat anti-Rabbit IgG Alexa-546(Invitrogen Cat# A-11010, RRID: AB_2534077) for 1 hr, nuclear staining was performed using DAPI solution (Sigma). Cells were imaged and analyzed using the Olympus FV1000 confocal Microscope (Olympus). Distribution of mitochondria across cytoplasm were carried out with the FV10-ASW 4.2 Viewer software. To quantify the radial distribution pattern of the mitochondria, we plotted the MitoTracker Red intensity in 2-3 μm wide lines that cover the clustered mitochondria starting from the nuclear envelope and radiating towards the cell periphery. Quantification of nuclear fluorescence intensity in tissues was performed in CellProfiler (version 4.2.1) using “MeasureObjectIntensity” module. CellProfiler pipelines will be provided upon request.

### Transmission electron microscopy

Cells were fixed with 2.5% glutaraldehyde solution (Sigma-Aldrich, Cat# G6257) in PBS (pH 7.4) at 4 °C for 4 hr, and post-fixed with 1% osmium tetroxide/PBS solution (Electron Microscopy Sciences, Cat# 19140) at room temperature for 1 hr. Samples were then washed with PBS (pH7.4) for 15 min for 3 times, dehydrated using series of ethanol to acetone solutions, infiltrated, and embedded in epoxy resin (Sigma-Aldrich, Cat# 31185, polymerized in 60 °C thermostats for 48 hr). Samples were cut into 70 nm thick sections on formvar-coated nickel slot grids, followed by staining with 2% uranyl acetate (Electron Microscopy Sciences, Cat# 22400) and 0.4% lead citrate (Sigma-Aldrich, Cat# 15326) at room temperature for 15 min. Sections were examined under the transmission electron microscope (Tecnai G2 Spirit BioTWIN).

### Immunohistochemical (IHC) and scoring

The application of human samples was approved by the Institutional Review Board (IRB) of the Sun Yat-sen University Cancer Center (#GZR2020-059). Formalin-fixed, paraffin-embedded human breast tissue samples were retrieved from the archives and the biobank of the Department of Pathology, Sun Yat-sen University Cancer Center. Immunohistochemical in paraffin-embedded tissues was performed as previously descried.^41^ Briefly, sectioned slides were deparaffinized in xylene and rehydrated. Antigenic retrieval was processed with sodium citrate. The sections were then incubated in H_2_O_2_ (3%) for 15 min, blocked in 10% goat serum for 60 min and incubated with α-phosATF1(ser63) antibody (1:1000, α-phospho-CREB1/ATF1, Abcam, Cat# ab76085, RRID: AB_1523174) at 4°C overnight. Secondary antibody, H_2_O_2_-diaminobenzidine, counterstained with hematoxylin, dehydrated, and mounted according to the manufacturer’s protocol (Dako). IHC scoring was evaluated by at least 2 independent pathologists. Scoring was performed by staining intensity and area follows: negative (score 0), bordering (score 1), weak (score 2), moderate (score 3), and strong (score 4).

### Statistical analysis

Statistical analyses were performed with SPSS software (version 16.0, RRID:SCR_002865) or GraphPad Prism 6.0 (GraphPad Software, RRID:SCR_002798). Results were expressed as mean ± SD, and the number of individual replicates. Statistical significance of the mean values was established by two-tailed distribution Student’s *t* test and ANOVA.

## Supplementary information

Supplementary Materials

Original and uncropped films of Western blots

Supplementary Table 1

Supplementary Table 2

Supplementary Table 4

Supplementary Table 5

Supplementary Movie S1

## Data Availability

Supplementary Figs. S1-9, Table S1-5 and Movies S1 are provided with the paper. The raw high throughput sequence data have been deposited in the Genome Sequence Archive, Beijing Institute of Genomics (BIG), Chinese Academy of Sciences, under accession numbers HRA000745 that are publicly accessible at https://bigd.big.ac.cn/gsa. All data supporting the findings are available from the corresponding author upon reasonable request.
