# Correction: Cancer cell employs a microenvironmental neural signal *trans*-activating nucleus-mitochondria coordination to acquire stemness

**DOI:** 10.1038/s41392-025-02389-3

**Published:** 2025-09-02

**Authors:** Bin He, Rui Gao, Shasha Lv, Ailin Chen, Junxiu Huang, Luoxuan Wang, Yunxiu Feng, Jiesi Feng, Bing Liu, Jie Lei, Bing Deng, Bin He, Bai Cui, Fei Peng, Min Yan, Zifeng Wang, Eric W-F Lam, Bilian Jin, Zhiming Shao, Yulong Li, Jianwei Jiao, Xi Wang, Quentin Liu

**Affiliations:** 1 State Key Laboratory of Oncology in South China, Sun Yat-sen University Cancer Center, Guangzhou, 510060 PR China; 2 Department of Medical Oncology, The Seventh Affiliated Hospital, Sun Yat-sen University, Shenzhen, 510275 PR China; 3 Institute of Cancer Stem Cell, Cancer Center, Dalian Medical University, Dalian, 116023 PR China; 4 Zhongshan School of Medicine, Sun Yat-sen University, Guangzhou, 510080 PR China; 5 State Key Laboratory of Membrane Biology, Peking University School of Life Sciences, Beijing, 100871 PR China; 6 Department of Surgery and Cancer, Imperial College London, London, W12 0NN UK; 7 Department of Breast Surgery, Precision Cancer Medicine Center, Fudan University Shanghai Cancer Center, Shanghai, 200032 PR China; 8 State Key Laboratory of Stem Cell and Reproductive Biology, Institute of Zoology, Chinese Academy of Sciences, Beijing, 100101 PR China

**Keywords:** Cancer microenvironment, Cancer stem cells, Genome informatics, Stem-cell niche

Correction to: *Signal Transduction and Targeted Therapy* 10.1038/s41392-023-01487-4, published online 19 July 2023

After online publication of the article,^1^ the authors noticed one inadvertent mistake occurred in Supplementary Fig. S5q that needs to be corrected. The correct text was provided as follows. The original article has been corrected.

During the collation of raw data, the authors identified an inadvertent duplication error in Supplementary Fig. S5q, Case#4 of the shATF1 group (Supplementary Fig. S5q, lower right panel), which required correction following the article’s online publication^1^. The authors have amended the image misplacement. Specifically, an oversight during image processing resulted in the unintended reuse of the same image of Case#3 shATF1 in the Case#4 shATF1 group. The authors previously used the correct raw images to make statistical analyses. Therefore, the right panel of Figure 3l is correct, and the correction in the lower right panel of Supplementary Fig. S5q, does not affect the interpretation or conclusions of this figure. Though this correction does not affect the science, data or conclusions of this article, the authors still regret this error. The correct data are provided as follows.

Incorrect Supplementary Fig. S5q:
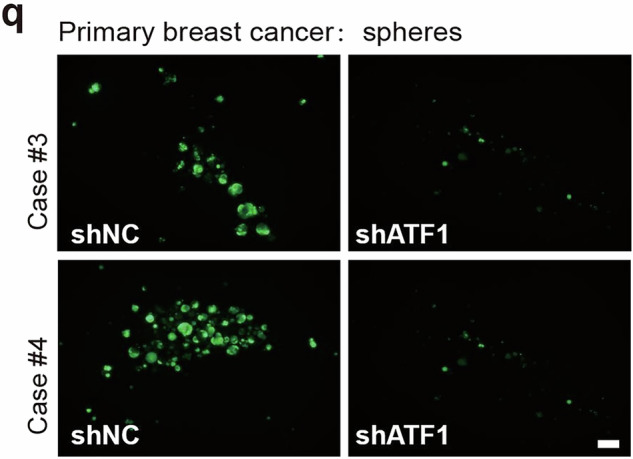


The revised Supplementary Fig.S5q is:
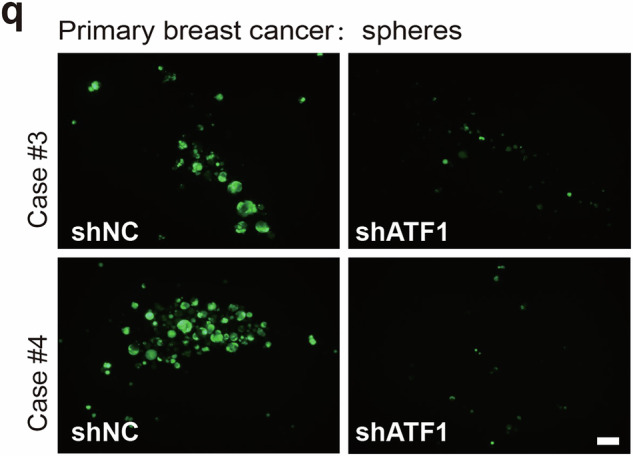


## Supplementary information

Supplementary Materials

Correct Supplementary Fig S5q
