# Supplementary material for: Cancer cell employs a microenvironmental neural signal trans-activating nucleus-mitochondria coordination to acquire stemness
Source: Signal Transduct Target Ther. 2023 Jul 19;8:275. doi: 10.1038/s41392-023-01487-4 (PMC10354099; doi:10.1038/s41392-023-01487-4)
Supplement: Supplementary file 1 — Supplementary Materials [file 41392_2023_1487_MOESM1_ESM.docx]

Supplementary Materials for

Cancer Cell Employs a Microenvironmental Neural Signal *Trans*-Activating Nucleus-mitochondria Coordination to Acquire Stemness

Bin He^1,9^, Rui Gao^1,2,9^, Shasha Lv^1,3,9^, Ailin Chen^1^, Junxiu Huang^4^, Luoxuan Wang^3^, Yunxiu Feng^3^, Jiesi Feng^5^, Bing Liu^1^, Jie Lei^1^, Bing Deng^1^, Bin He^3^, Bai Cui^3^, Fei Peng^3^, Min Yan^1^, Zifeng Wang^1^, Eric W-F Lam^6^, Bilian Jin^3^, Zhiming Shao^7^, Yulong Li^5^, Jianwei Jiao^8^, Xi Wang^1*^, and Quentin Liu^1,2,3*^

Correspondence to: liuq9@mail.sysu.edu.cn

**This PDF file includes:**

Figures. S1 to S9

Captions for Tables S1-2, S4-5

Tables S3

Captions for Movies S1

**Other Supplementary Materials for this manuscript include the following:**

Supplementary Tables S1-2, S4-5

Movies S1

Figure. S1.

**Supplementary Fig. S1. CRE is a conserved transcriptional regulator of CSCs. (a)** Correlations between StemnessScores and microenvironmental cell intensity in TCGA 33 cancer types as revealed by single sample GSEA (ssGSEA) of cell-type specific gene sets. Stem cells (red), neural cells (orange) and immune cells (blue) were labeled. **(b)** Pearson correlation between 30 paired *in vivo* tumorigenic frequency (1/tumorigenic cell frequency, N) and *in vitro* sphere formation frequency (1/tumorigenic cell frequency, n). **(c-d)** Representative enrichment plots for glioma stem cell **(c)** and transcription factor **(d)** gene sets in paired sphere-adherent datasets. Normalized enrichment scores (NES) and *P* values were determined by gene set enrichment analysis (GSEA). **(e)** The frequencies (x axis) and median NES (y axis) of transcription factors enriched in 15 paired sphere-adherent datasets. The cAMP responsive element (CRE) binding transcription factors were labeled in red.

Figure. S2.

**Supplementary Fig. S2. CRE activity is required for cancer stemness.** **(a)** Flow cytometry analysis of CRE-dGFP reporter in T47D cells treated with cAMP response activators (Butyl-cAMP, 5μM; Forskolin, 5μM) or inhibitor (SQ22536, 1μM) for 24 hr. dGFP, destabilized GFP. **(b)** Left panel, CRE-dGFP and CMV-dsRed activities of DLD1 and MDA-MB-231 cells in adherent and sphere cultures (day 2 and day 6 in sphere culture). Right panel, percentage of CRE-dGFP^+^ cells in adherent and sphere cultures (day 2 and day 6 in sphere culture, n = 3; mean ± SD; *P* values, Tukey’s multiple comparisons after 1-way ANOVA). **(c)** *In vitro* limit dilution assay of sorted CRE-dGFP^+^ and CRE-dGFP^-^ populations in MDA-H460 and DLD1 cells. Differences in stem cell frequencies were determined by ELDA (https://bioinf.wehi.edu.au/software/elda/). n = 6 for each group. **(d)** Western blot analysis of MDA-MB-231 cells treated with cAMP mimics (Butyl-cAMP and 8-Br-cAMP) for 1 hr. Cell extracts were analyzed with phospho-CREB1/ATF1, total ATF1/CREB1 and Alpha-tubulin antibodies. **(e)** Sphere formation of BT-549 cells treated with different doses of cAMP mimics (Butyl-cAMP and 8-Br-cAMP). **(f)** Proliferation of MDA-MB-231 and BT-549 cells in response to different doses of Butyl-cAMP. **(g)** Sphere formation of DLD1 and A549 cells treated with 5 μM of cAMP mimics (*P* values for figures **S2e-g**, Tukey’s multiple comparisons after 1-way ANOVA). (**h**) Western blot analysis of A549 (upper panel) and T47D (lower panel) cells treated with PKA inhibitor H89 for 1 hr. Cell extracts were analyzed with phospho-CREB1/ATF1, total ATF1/CREB1 and Alpha-tubulin antibodies. **(i)** Sphere formation of T47D, MCF-7, BT-549 and A549 cells in response to H89 in sphere media (n = 3 for, mean ± SD; *P* values, Tukey’s multiple comparisons after 1-way ANOVA). **(j)** Representative images of sphere formation in BT-549 (upper panel) and T47D (lower panel) cells treated with H89. Scale bar, 100 μm. **(k)** Proliferation of MDA-MB-231, T47D and DLD1 cells in response to 5 μM H89 and DMSO (n = 3; mean ± SD; *P* values, Tukey’s multiple comparisons after 1-way ANOVA). **(l-m)** Western blot analysis of MDA-MB-231 (**l**), BT-549 and A549 (**m**) cells treated with PKA inhibitor (Metadoxine) and MSK1/2 inhibitor (SB-747651A) for 1 hr. Cell extracts were analyzed with phospho-CREB1/ATF1, total CREB1/ATF1 and Alpha-tubulin antibodies. **(n)** Sphere formation of MDA-MB-231, BT549 and A549 cells treated with indicated doses of inhibitors (*P* values, Tukey’s multiple comparisons after 1-way ANOVA). *, *P* < 0.05; **, *P* < 0.01; ***, *P* < 0.001; ****, *P* < 0.0001.

Figure. S3.

**Supplementary Fig. S3. Deconvolution of neural signals in human cancer. (a)** Heatmap showing the expressions of neural specific genes in GTEX dataset. Sidebar, tissue of origin. **(b)** Representative tSNE plots showing the expression of individual neural specific genes (TH, ACHE, SLC18A2) in single cells from breast cancer, melanoma, cervical cancer, pancreatic cancer, glioma and medulloblastoma. **(c)** Expression of SYP (left panel) and SNAP25 (right panel) in pan-TCGA tumors grouped by tumor stages (*P* values, Wilcoxon test). **(d)** Kaplan Meier analysis of disease-free survival (DFS) in pan-TCGA cancer patients with low (blue curve) and high (red curve) expression of SYP (left panel) and SNAP25 (right panel). Pan-cancer data in **S3c-d** were analyzed in the GEPIA database (http://gepia.cancer-pku.cn/index.html). **(e)** The neural infiltrating states of normal (GTEX dataset) tissues according to the ssGSEA enrichment scores. **(f)** Correlation of CRE activity with neural genes in the 31 TCGA and METABRIC breast tumors. Correlations (Pearson *r*) between CRE and individual neural signals was determined according to their ssGSEA scores in individual tumors.

Figure. S4.

**Supplementary Fig. S4. Cancer associated norepinephrine activate CRE dependent stemness.** **(a)** GSEA enrichment of transcription factor gene sets enriched in depressed ovarian cancer patients (GSE9116). CRE factors were labeled in red. **(b)** GSEA enrichment plots for stemness gene sets enriched in GSE9116. Patients were grouped according to their documented depression levels. **(c)** Representative immunohistochemical (IHC) staining of phospho-CREB1/ATF1 in MDA-MB-231 xenografts from control (Ctrl) and stressed mice. Scale bar, 100 μm. **(d)** Western blot analysis of total lysates from MDA-MB-231 (upper panel) and T47D (lower panel) cells treated with Glutamate (Glu) and Acetylcholine (Ach) for 30 min. **(e)** Western blot analysis of total lysates from DLD1 (upper panel) and H460 (lower panel) cells treated with epinephrine (EP) and norepinephrine (NE) for 30 min. **(f)** Percentage of CRE-dGFP^+^ cells in T47D and DLD1 cells in response to NE/EP treatment (24 hr, n = 3; mean ± SD). **(g-i)** Sphere formation of MDA-MB-231 **(g)**, T47D **(h)**, H460 and DLD1 **(i)** cells in the presence of NE/EP (n = 3; mean ± SD; *P* values for figures **S4f**-**i**, Tukey’s multiple comparisons after 1-way ANOVA). Representative images of spheres were present. Scale bar, 60 μm. **(j)** Western blot analysis of total lysates from T47D (upper panel) and MDA-MB-231 (lower panel) cells treated with indicated doses of epinephrine (EP) and norepinephrine (NE) for 30 minutes and 24 hours. **(k)** Fluorescent images of MDA-MB-231 cells expressing norepinephrine reporters (NE2m and NE2h, green) and tdTomato (red). Cells were treated with norepinephrine (5μM) for 5 min before imaging. Scale bar, 20 μm. **(l)** Quantification of norepinephrine reporter intensity (median fluorescent intensity quantified with CellProfiler) in MDA-MB-231 reporter cells. The ratio of NE2h/tdTomato for individual cells were calculated (*P* values, Mann Whitney test). **(m)** Representative images of GRAB_NE2h_ (green) and tdTomato (red) activity in the MDA-MB-231 xenograft. White arrows indicate cells with high NE2h activity. Scale bar, 60 μm. **(n)** QRT-PCR (QPCR) validation of ADRB2 in iDox-shARDB2 MDA-MB-231 cells. **(o)** Sphere formation of MDA-MB-231 and BT-549 cells expressing iDox-shADRB2. Dox+ cells were treated with Dox for 4 days prior to sphere formation assay (*P* values, Tukey’s multiple comparisons after 1-way ANOVA). **(p)** Left panel, *in vivo* limit dilution assay of MDA-MB-231 cells expressing iDox-shADRB2 in NSG mice treated with or without Dox in drinking water. Differences in stem cell frequencies were determined by ELDA (https://bioinf.wehi.edu.au/software/elda/). n = 5 for each group, respectively. Right panel, Western blot analysis of total lysates from control and Dox groups of MDA-MB-231-iDox-shADRB2 xenografts. *, *P* < 0.05; **, *P* < 0.01; ***, *P* < 0.001; ****, *P* < 0.0001.

Figure. S5.


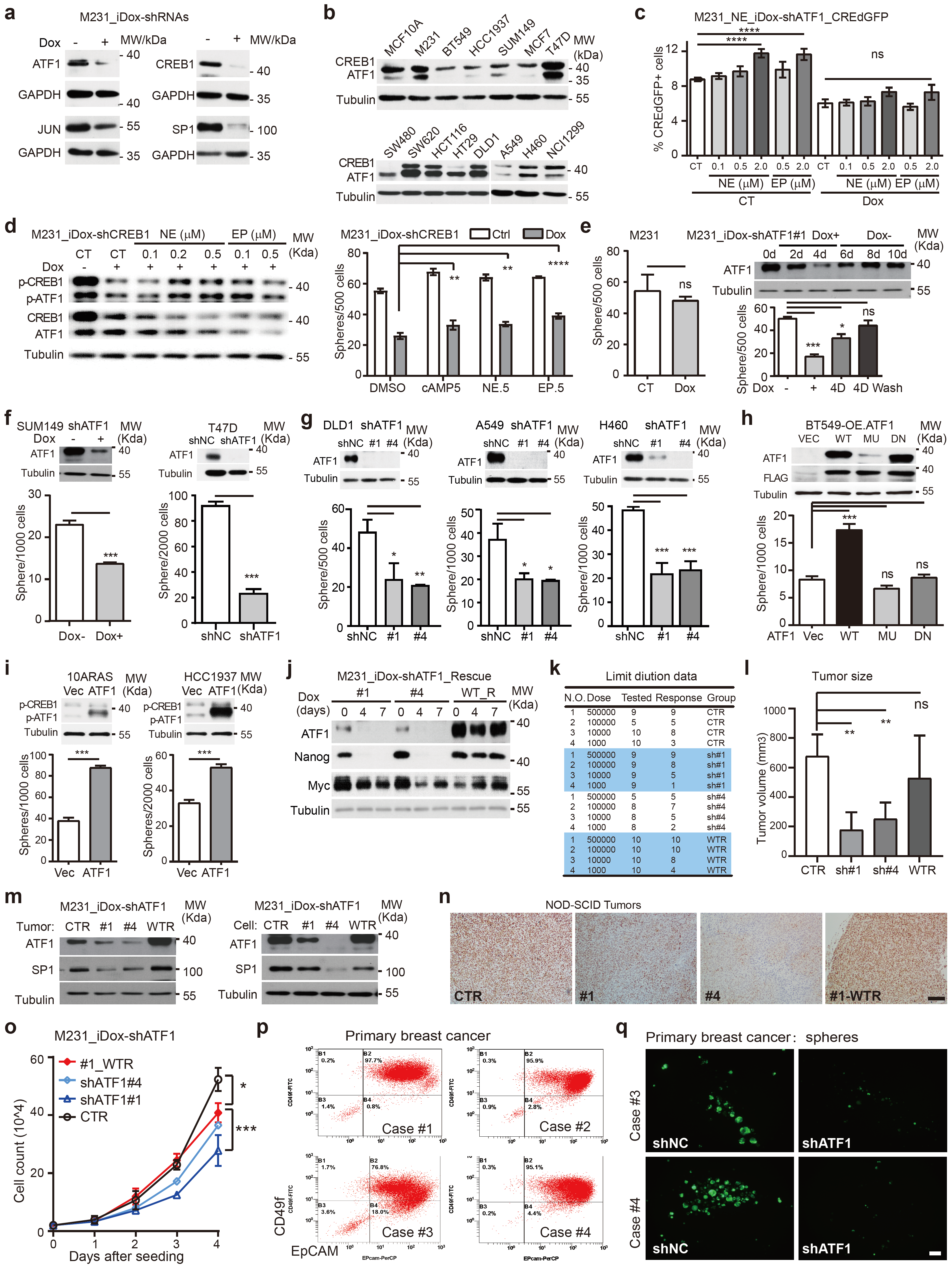


**Supplementary Fig. S5. ATF1 dictates neural signals to potentiate cancer stemness. (a)** Western blot analysis of MDA-MB-231 cells expressing iDox-shRNAs against ATF1, CREB1, JUN and SP1. Cell extracts were analyzed with ATF1, CREB1, JUN, SP1 and GAPDH antibodies. **(b)** Western blot analysis of human cell lines. Cell extracts were analyzed with ATF1/CREB1. Alpha-tubulin served as the loading control. **(c)** Percentage of CRE-dGFP^+^ cells in iDox-shATF1 MDA-MB-231, Cells with/without Dox treatment were determined in response to NE/EP treatment (24 hr, n = 3; mean ± SD; *P* values, Tukey’s multiple comparisons after 1-way ANOVA). **(d)** Left panel, Western blot analysis of shCREB1 cells in respond to epinephrine and norepinephrine. Right panel, MDA-MB-231 iDox-shCREB1 cells treated with Dox in combination with DMSO, 8Br-cAMP (5 μM) and epinephrine (EP, 0.5 μM) and norepinephrine (NE, 0.5 μM) in sphere assays (n = 3; mean ± SD; *P* values, Tukey’s multiple comparisons after 1-way ANOVA). **(e)** Sphere formation of MDA-MB-231 cells treated with Dox or PBS control (CT left panel, n = 3; mean ± SD; *P* values, 2-sided *t* test). Western blot analysis and sphere formation of iDox-shATF1#1 MDA-MB-231 cells (right panel, n = 3; mean ± SD; *P* values, Tukey’s multiple comparisons after 1-way ANOVA). **(f)** Western blot and sphere formation analyses of iDox-shATF1 SUM149 (left panel) and T47D (right panel) breast cancer cells in CT/Dox media, respectively (n = 3; mean ± SD; *P* values, 2-sided *t* test). **(g)** Western blot and sphere formation analyses of control (shNC) and shATF1 cells of DLD1 (left panel), A549 (middle panel) and T47D (right panel), respectively. **(h)** Western blot analysis of BT-549 cells expressing vector (Vec) or different forms of ATF1(wild type (WT), S63A (MU) and R231L (DN), left panel). Sphere formation of BT549 cells expressing different forms of ATF1 (right panel). For figures **S5g-h**, n = 3; mean ± SD; *P* values, Tukey’s multiple comparisons after 1-way ANOVA). **(i)** Western blot analysis of HCC1937 and MCF10A-HRAS^G12V^ cells expressing vector (Vec) or ATF1(wild type, upper panel). Sphere formation of HCC1937 and MCF10A-HRAS^G12V^ cells expressing ATF1 or vector control (lower panel). n = 3; mean ± SD; 2-sided *t* test. **(j)** Western blot analysis of iDox-shATF1#1, #4 and wild-type ATF1 rescued MDA-MB-231 cells treated with Dox for 4 and 7 days. Cell extracts were analyzed with ATF1, NANOG and MYC antibodies. Alpha-tubulin served as the loading control. **(k)** Limiting dilution transplantation of iDox-shATF1 or ATF1 rescued MDA-MB-231 cells in NOD-SCID mice. The number of established tumors and the number of animals transplanted. **(l)** Tumor volume of iDox-shATF1 or ATF1 rescued MDA-MB-231 cells in NOD-SCID mice (group: 5 × 10^^5^ cells, *P* values, Tukey’s multiple comparisons after 1-way ANOVA). **(m)** Western blot analysis of MDA-MB-231 xenografts tumors or tumor-derived cells. Antibodies against ATF1 and SP1 and Alpha-tubulin were applied. **(n)** Representative IHC staining of ATF1/CREB1 in different groups of MDA-MB-231 derived xenografts from figure **S5k**. Scale bar, 50 μm. **(o)** Proliferation of tumor-derived MDA-MB-231 cell from **S5k** (n = 3; mean ± SD; *P* values, Tukey’s multiple comparisons after 1-way ANOVA). **(p)** Representative flow cytometry of primary breast cancer cells derived from four patients (Case #1 - #4). Cells were stained with antibodies against mammary epithelial markers: CD49f-FITC and EpCAM-PerCP. **(q)** Representative images indicating sphere formation from **Fig. 3l**. Scale bar, 100 μm. *, *P* < 0.05; **, *P* < 0.01; ***, *P* < 0.001; ****, *P* < 0.0001; ns, not significant.

Figure. S6.

**Supplementary Fig. S6. ATF1 *trans*-activates pluripotent and mitochondrial regulators. (a)** Gene Ontology annotation of upregulated (upper panel) and down-regulated (lower panel) genes in shATF1 group (overlapping genes between shATF1#1 and shATF1#4) compared to control MDA-MB-231 cells. Gene lists were uploaded and analyzed in the DAVID database. Top biological processes (BP), cellular component (CC) and molecular function (MF) terms were included. **(b)** QRT-PCR (QPCR) validation of candidate differentially expressed genes (list from RNA-seq analysis) in iDox-shATF1 MDA-MB-231 cells. **(c)** QPCR analysis of genes involved in mitochondrial dynamics and bioenergetics in iDox-shATF1#1 MDA-MB-231 cells. Fold changes (presented as Log2) in gene expression were compared to cells without Dox treatment (n = 3; mean ± SD). **(d)** Representative tracks of normalized ATF1 CUT&TAG-seq signals. **(e)** Pearson correlations among transcription factor and stemness scores. Scores were determined by ssGSEA based on the gene expression profiles of 1019 cancer cell lines from the Cancer Cell Line Encyclopedia (CCLE). **(f)** Representative plots of enriched cellular senescence associated gene sets in iDox-shATF1 (shATF1#1 and shATF1#4) cells. **(g)** Cell proliferation of iDox-shATF1 MDA-MB-231 cells (n = 3; mean ± SD; *P* values, Tukey’s multiple comparisons after 1-way ANOVA). **(h)** Representative results for colony formation of iDox-shATF1#1/#4 MDA-MB-231 cells. Cells were seeded in 6-well plates for 3 weeks before crystal violet staining. Scale bar, 5mm. **(i)** Flow cytometry analysis of apoptosis by PI and Annexin-V-FITC staining. The iDox-shNC/shATF1#1/#4 MDA-MB-231 cells were treated with Dox for 6 days. The amounts of apoptotic (Annexin^+^, marked in red box) cells were displayed on the right panel (n = 3; mean ± SD; *P* values, Tukey’s multiple comparisons after 1-way ANOVA). **(j)** The iDox-shATF1 or wild-type ATF1 rescued MDA-MB-231 cells were treated with Dox for 7 days, fixed and stained for SA-β-Gal analysis. The percentage of SA-β-Gal^+^ cells was shown on the right. Scale bar, 20 μm. (n = 3; mean ± SD; *P* values, Tukey’s multiple comparisons after 1-way ANOVA). **(k)** Western blot analysis of iDox-shATF1#1 MDA-MB-231 cells. Lysates were analyzed with indicated antibodies (n = 3; representative data). **(l)** The iDox-shATF1#1 MDA-MB-231 cells were labeled with PKH26 4 days before Dox treatment. Cells were harvested after 7 days of Dox treatment, fixed and stained for SA-β-Gal. The percentage of SA-β-Gal^+^ cells was shown on the lower panel. Scale bar, 20 μm (n = 3; mean ± SD). **(m)** The iDox-shATF1#1 MDA-MB-231 cells in sphere cultures at day 6 were fixed and stained for SA-β-Gal. Scale bar, 60 μm (n = 3; mean ± SD; *P* values, Chi-square test). *, *P* < 0.05; **, *P* < 0.01; ***, *P* < 0.001; ****, *P* < 0.0001; ns, not significant.

Figure. S7.

**Supplementary Fig. S7. ATF1 depletion impairs mitochondrial rejuvenation.** **(a)** Mitochondrial mass as determined by flow cytometry-based Mitotracker intensity in iDox-shATF1/shGFP MDA-MB-231 cells treated with Dox for 2, 4 and 6 days, respectively (n = 3; mean ± SD; *P* values, Tukey’s multiple comparisons after 1-way ANOVA). **(b)** Mitochondrial ROS (mROS) in A549 cells determined by flow cytometry using MitoSOX Red. Cells were stained with MitoSOX Red for 15 min (n = 3; mean ± SD; 2-sided *t* test). **(c)** Mitochondrial membrane potential as indicated by flow cytometry using Rhodamine 123. The iDox-shATF1#1/#4 and parental MDA-MB-231 cells were treated with Dox for 4 days (n = 3; mean ± SD; *P* values, Tukey’s multiple comparisons after 1-way ANOVA). **(d)** Mitochondrial membrane potential determined by flow cytometry using Rhodamine 123 staining in iDox-shATF1 A549 cells (CT/Dox for 4 days; n = 3; mean ± SD; 2-sided *t* test). For **S7a**-**d,** the changes in median fluorescent intensity (MFI) were compared to those of control group. **(e)** Representative plot showing the mitochondrial turnover reporter mitoTimer determined by flow cytometry in iDox-shATF1#1/#4 MDA-MB-231, iDox-shATF1 T47D, A549 and H460 cells. Cells were analyzed 48 hr after transiently transfected with mitoTimer. Cells expressing GFP were overlapped as controls to distinguish GFP and RFP signals. **(f)** Representative plots showing mitochondrial damage determined by flow cytometry of MitoTracker Deep-Red and MitoTracker Green. Parental MDA-MB-231 or iDox-shGFP cells were treated with Dox for 4 days, challenged with CCCP for 6h before MitoTracker staining. **(g)** Percentages of cells with damaged mitochondria (MitoTracker Deep-Red^low^ Green^high^) cells in **S7f** (n = 3; mean ± SD; *P* values, Tukey’s multiple comparisons after 1-way ANOVA). **(h)** Western blot analysis of iDox-shATF1 MDA-MB-231 cells treated with combinations of Dox, CCCP and dynein inhibitor (DynI). Lysates were analyzed with ATF1, LC3, p62, Tom20 and GAPDH antibodies. **(i)** Percentages of cells with damaged mitochondria. Cells were treated with CCCP in combination with lysosome inhibitor (Leup). n = 3; mean ± SD; *P* values, Tukey’s multiple comparisons after 1-way ANOVA. **(j)** Mitochondria localization determined by confocal analysis using MitoTracker Red staining. MDA-MB-231 cells were transfected with siRNAs against NC and siATF1 RNA. MitoTracker Red intensity (mean ± SD) as a function of distance to nuclei was analyzed (n = 20 for each group, respectively). **(k-m)** MDA-MB-231 cells **(k)**, iDox-shATF1 A549 **(l)** and iDox-shATF1 T47D **(m)** cells were treated with Dox for 4 days before staining. MitoTracker Red intensity (mean ± SD) as a function of distance to nuclei was analyzed (n = 20 for each group, respectively). **(n)** Sphere formation of iDox-shATF1#1 MDA-MB-231 cells. Cells expressing vector (Vec) or dynein inhibitory protein DCTN2 were treated with/without Dox. Cells were co-treated Dox and Dynein inhibitor (2-20 μM) and analyzed in sphere formation (n = 3; mean ± SD; *P* values, Tukey’s multiple comparisons after 1-way ANOVA). **(o)** MitoTracker Red staining in iDox-shATF1 MDA-MB-231 cells treated with Dox alone, or in combination with MitoQ. Scale bar, 20 μm (n = 3; mean ± SD). **(p)** Mitochondrial membrane potential determined by Rhodamine 123 in iDox-shATF1 MDA-MB-231 cells treated with Dox alone, or in combination with mitoTempo (MitoT) or MitoQ (μM, n = 3; mean ± SD; *P* values, Tukey’s multiple comparisons after 1-way ANOVA). **(q)** Representative flow cytometry plots for ALDEFLUOR activity in iDox-shATF1 MDA-MB-231 cells treated with Dox alone, or in combination with mitoTempo (MitoT) or MitoQ (μM). **(r)** ALDEFLUOR activity in iDox-shATF1 MDA-MB-231 cells treated with mitoTempo (MitoT) or MitoQ (n = 3; mean ± SD; *P* values, Tukey’s multiple comparisons after 1-way ANOVA). *, *P* < 0.05; **, *P* < 0.01; ***, *P* < 0.001; ****, *P* < 0.0001; ns, not significant.

Figure. S8.

**Supplementary Fig. S8. Coordinated nucleus-mitochondria program in cancer stem-like state.** **(a)** Mitochondrial membrane potential as determined by Rhodamine 123 in MDA-MB-231, A549 and DLD1 cells in adherent (Adh) or suspension (Sus) culture for one day. **(b)** Rhodamine 123 intensity in shNC/shATF1 MDA-MB-231 (left panel) and T47D (right panel) cells seeded in adherent (Adh) or suspension (Sus) culture for one day (for figures **S8a-b**, n = 3; mean ± SD; *P* values, Tukey’s multiple comparisons after 1-way ANOVA). **(c)** Representative plots NANOG- or MYC-promoter activity using promoter-dGFP reporter cell lines. Cells were treated Dox for 3 days, seeded in adherent or suspension culture for one day, followed by flow cytometry analysis. **(d)** Western blot of iDox-shATF1 MDA-MB-231 cells rescued with Dox inducible expression of NRF1, NANOG, SOX2 and MYC, respectively. Cells lysates were analyzed with Flag and tubulin antibodies. **(e)** Left panel, Western blot analysis of iDox-shATF1 A549 rescued with Dox inducible expression of NRF1, NANOG and MYC, respectively. Cells lysate were analyzed with indicated antibodies. Right panel, sphere formation of iDox-shATF1 A549 cells rescued with Dox inducible NRF1, NANOG or MYC (n = 3; mean ± SD; *P* values, Tukey’s multiple comparisons after 1-way ANOVA). **(f)** Left panel, Western blot and representative images of sphere formation in rescued iDox-shATF1 H460 cells. Right panel, sphere formation iDox-shATF1 H460 cells rescued with NRF1 and MYC, respectively (n = 3; mean ± SD; *P* values, Tukey’s multiple comparisons after 1-way ANOVA). Scale bar, 60 μm. **(g)** QPCR analyses of stemness genes and mitochondrial biogenesis genes in iDox-shATF1 MDA-MB-231 cells rescued with NRF1, NANOG, SOX2 and MYC, respectively (n = 3; mean ± SD). **(h)** QPCR analyses of mitochondrial biogenesis genes in iDox-shATF1 MDA-MB-231 and A549 cells rescued with MYC or vector controls, respectively (n = 3; mean ± SD). **(i)** Mitochondrial ROS (mSOX) of rescued iDox-shATF1 MDA-MB-231 cells as determined by MitoSOX (n = 3; mean ± SD; *P* values, Tukey’s multiple comparisons after 1-way ANOVA). **(j)** Representative mitochondria localization determined by MitoTracker Red staining in iDox-shATF1 MDA-MB-231 cells rescued with candidate transcription factors (NRF1, MYC). Scale bar, 20 μm. **(k)** Left panel, mitochondrial membrane potential of rescued iDox-shATF1 MDA-MB-231 cells as determined by Rhodamine 123. Right panel, MFI for Rhodamine intensities in MDA-MB-231 cells. **(l)** The iDox-shATF1 A549 and H460 cells rescued with ectopic expression vectors (NANOG, MYC, NRF1) were stained with Rhodamine 123. MFI for Rhodamine intensities were analyzed (for figures **S8k-l**, n = 3; mean ± SD; *P* values, Tukey’s multiple comparisons after 1-way ANOVA). **(m)** Left panel, Western blot of iDox-shNRF1 and iDox-shNANOG MDA-MB-231 cells. Cells treated with Dox for 4 days were compared with the Dox-free cells. Right panel, sphere formation of iDox-shNRF1/shNANOG MDA-MB-231 cells with/without Dox treatment. **(n)** Mitochondrial turnover as determined by flow cytometry analysis of mitoTimer in iDox-shNRF1 and iDox-shNANOG MDA-MB-231 cells with/without Dox. **(o)** Mitochondrial membrane potential (left panel) and mitochondrial ROS (right) as determined by flow cytometry analysis with Rhodamine 123 and MitoSOX Red, respectively. (Figures **S8m-o**, n = 3; mean ± SD; *P* values, Tukey’s multiple comparisons after 1-way ANOVA). **(p-q)** Kaplan Meier analysis of relapse-free survival (RFS, **S8p**) and overall survival (OS, **S8q**) in patients with low (black curve) and high (red curve) expression gene signatures. ATF1 targeting (motif-based targets listed in Supplementary Table 2) pluripotent genes (Nucl, upper panel), mitochondrial genes (Mito, middle panel) or a combination of pluripotent and mitochondrial genes (Comb, lower panel) gene signatures were assessed in the Kaplan Meier plotter datasets (http://kmplot.com/analysis/index.php?p=background). *, *P* < 0.05; **, *P* < 0.01; ***, *P* < 0.001; ****, *P* < 0.0001; ns, not significant.

Figure. S9.

**Supplementary Fig. S9. Single-cell based nucleus-mitochondria coordination in CSCs. (a)** Pearson correlation between CRE transcription factor and stemness scores. Scores were determined by ssGSEA based on the single-cell transcriptome datasets. **(b-d)** The ssGSEA enrichment scores of stemness (ESC), nuclear transcription factor (MYC, NANOG) and mitochondrial gene sets based on the single-cell transcriptome of cancer cells. Lung cancer (GSE136580) and skin squamous cell carcinoma (SCC) cells (GSE108679) were grouped by CSC markers (NE in GSE136580, CD44/CD34/ITGA6 in GSE108679) in **S9b**. Acute myeloid leukemia (AML, GSE140896) and breast cancer CSCs (CD24^-^CD44^+^, GSE124887) datasets were displayed in **S9c**. The ssGSEA enrichment scores of ESC, MYC and mitochondrial gene sets were shown in **S9d**. **(e)** The single-cell ssGSEA enrichment scores of stemness (ESC), nuclear transcription factor (MYC, NANOG) and mitochondrial gene sets based on the single-cell transcriptome of normal and cancer cells. Glioma stem cell (GSC, GSE132172), CML stem cell (LSC, GSE81730) and breast cancer cells (GSE138536) were compared with normal cells within the same tissues, respectively. For **S9e**, Pearson correlations between scores were determined in normal and cancer subsets, respectively. **(f-g)** The ssGSEA enrichment scores of stemness (ESC), nuclear transcription factors (NANOG, MYC) and mitochondrial gene sets in single normal and cancer cells (2-sided *t* test) **(h)** Correlation between StemnessScores and gene sets involved in stemness, mitochondria and nuclear pluripotent factors in the 33 cancer types from the TCGA dataset. Machine learning StemnessScores and ssGSEA scores of individual tumors were analyzed (Pearson *r*). *, *P* < 0.05; **, *P* < 0.01; ***, *P* < 0.001; ****, *P* < 0.0001.

Table S1 (Separate file).

Stem factors enriched in tumor or cell models.

Table S2 (Separate file).

Gene lists for prognostic analysis.

Table S3.

Correlation between phospho-CREB1/ATF1 and clinical parameters.

|  | ATF1 positive | | ATF1 negative | |  |
| --- | --- | --- | --- | --- | --- |
| Factor | NO. | % | NO. | % | *P* value |
| All patients | 186 | 50.7 | 181 | 49.3 |  |
| **Age (years)** |  |  |  |  | 0.923 |
| ≤50 | 116 | 50.9 | 112 | 49.1 |  |
| >50 | 70 | 50.4 | 69 | 49.6 |  |
| **Menopauses** |  |  |  |  | 0.563 |
| Perimenopauses | 119 | 49.6 | 121 | 50.4 |  |
| Perimenopauses | 67 | 52.8 | 60 | 47.2 |  |
| **Tumor size (cm)** |  |  |  |  | 0.134 |
| ≤2 | 61 | 45.5 | 73 | 54.5 |  |
| >2 | 125 | 53.6 | 108 | 46.4 |  |
| **Node status** |  |  |  |  | 0.089 |
| Negative | 72 | 45.6 | 86 | 54.4 |  |
| Positive | 114 | 54.5 | 95 | 45.5 |  |
| **Tumor grade** |  |  |  |  | 0.036* |
| 1 or 2 | 67 | 59.8 | 45 | 40.2 |  |
| 3 | 31 | 52.5 | 28 | 47.5 |  |
| Missing | 88 |  | 108 |  |  |
| **Stage** |  |  |  |  | 0.234 |
| I or II | 109 | 48.2 | 117 | 51.8 |  |
| III | 77 | 54.6 | 64 | 45.4 |  |
| **ER status** |  |  |  |  | 0.110 |
| Negative | 15 | 38.5 | 24 | 61.5 |  |
| Positive | 170 | 52.0 | 157 | 48.0 |  |
| Missing | 1 |  |  |  |  |
| **PR status** |  |  |  |  | 0.044* |
| Negative | 20 | 37.7 | 33 | 62.3 |  |
| Positive | 165 | 52.7 | 148 | 47.3 |  |
| Missing | 1 |  |  |  |  |
| **HER2 status** |  |  |  |  | 0.020* |
| Negative | 118 | 57.6 | 87 | 42.4 |  |
| Positive | 67 | 41.6 | 94 | 58.4 |  |
| **Histology type** |  |  |  |  | 0.438 |
| Invasive ductal | 171 | 49.9 | 172 | 50.1 |  |
| Other | 10 | 41.7 | 14 | 58.3 |  |
| **Distant Metastasis** |  |  |  |  | 0.002* |
| Negative | 138 | 46.8 | 157 | 53.2 |  |
| Positive | 48 | 66.7 | 24 | 33.3 |  |

Table S4 (Separate file).

Primers for RNAi (Table S4a), QPCR (Table S4b) and ChIP-PCR (Table S4c).

Table S5 (Separate file).

Differentially expressed gene lists in RNA-seq groups of MDA-MB-231 cells expressing iDox-shATF1/shCREB1.

Movie S1 (Separate file).

Time-lapse imaging of fluorescent norepinephrine reporter (NE2h, Green signal) activity in breast cancer xenograft. MDA-MB-231 cells expressing a NE2h-T2A-tdTomato vector were implanted subcutaneously. Tumor-bearing mouse was imaged for 20 minutes after i.p. injection of the NET blocker desipramine (10 mg/kg, middle). Red signal indicates tdTomato. Scale bar, 60 μm.
