# Supplementary material for: Cancer cell employs a microenvironmental neural signal trans-activating nucleus-mitochondria coordination to acquire stemness
Source: Signal Transduct Target Ther. 2023 Jul 19;8:275. doi: 10.1038/s41392-023-01487-4 (PMC10354099; doi:10.1038/s41392-023-01487-4)
Supplement: Supplementary file 2 — Original and uncropped films of Western blots [file 41392_2023_1487_MOESM2_ESM.docx]

Original and uncropped films of Western blots
