# Supplementary figures and images for: Correction: Cancer cell employs a microenvironmental neural signal trans-activating nucleus-mitochondria coordination to acquire stemness
Source: Signal Transduct Target Ther. 2025 Sep 2;10:289. doi: 10.1038/s41392-025-02389-3 (PMC12405508; doi:10.1038/s41392-025-02389-3)

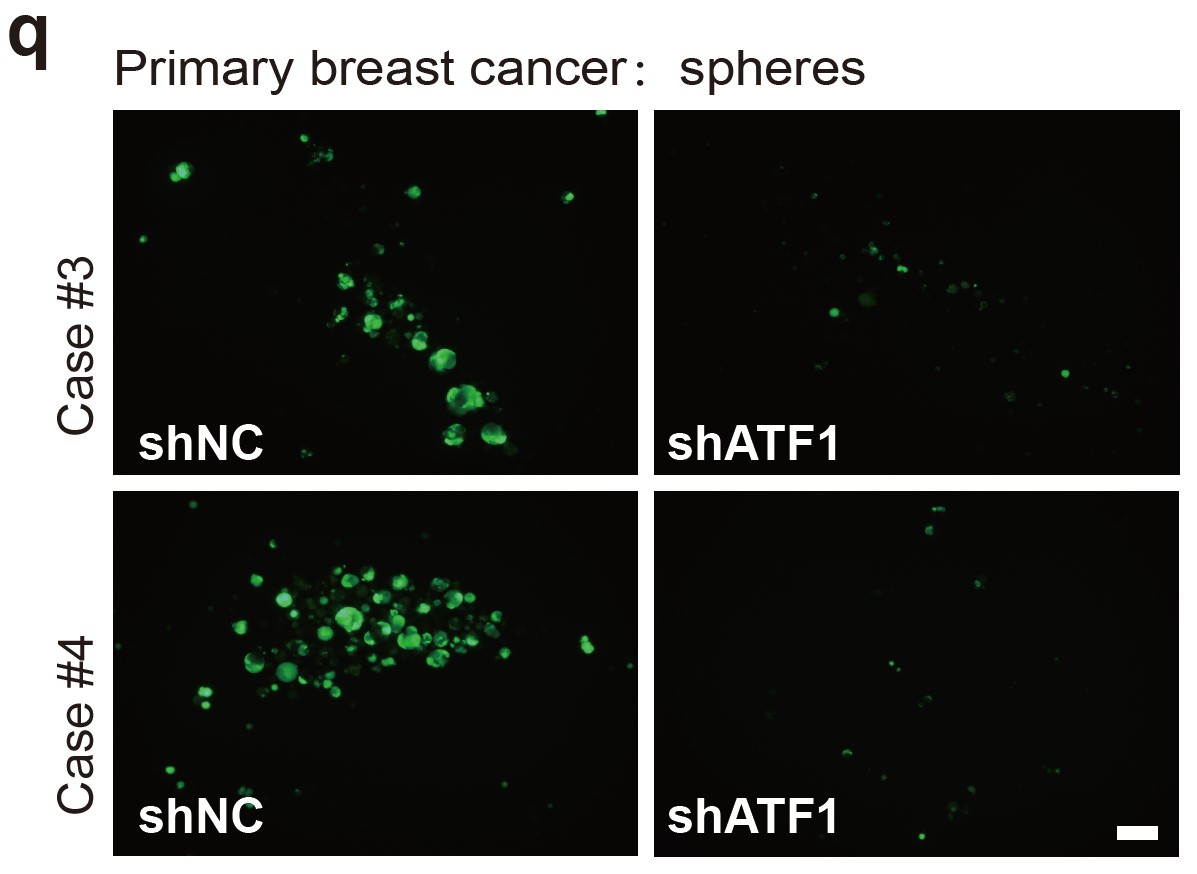

Supplement: Supplementary file 2 — Correct Supplementary Fig S5q [file 41392_2025_2389_MOESM2_ESM.jpg]
